# The emerging role of deubiquitylating enzymes as therapeutic targets in cancer metabolism

**DOI:** 10.1186/s12935-022-02524-y

**Published:** 2022-03-20

**Authors:** Rongfu Tu, Junpeng Ma, Peng Zhang, Ye Kang, Xiaofan Xiong, Junsheng Zhu, Miao Li, Chengsheng Zhang

**Affiliations:** 1 Precision Medicine Center, The First Affiliated Hospital of Xi’an Jiaotong University, 277 Yanta West Road, Xi’an, 710061 China; 2 Department of Cancer Precision Medicine, The MED-X Institute, The First Affiliated Hospital of Xi’an Jiaotong University, Building 21, Western China Science and Technology Innovation Harbor, Xi’an, 710000 China; 3 Cancer Center, The First Affiliated Hospital of Xi’an Jiaotong University, 277 Yanta West Road, Xi’an, 710061 China; 4 Department of Tumor and Immunology, Precision Medicine Institute, Western China Science and Technology Innovation Harbor, Xi’an, 710004 China; 5 The Jackson Laboratory for Genomic Medicine, Farmington, CT 06032 USA

**Keywords:** Deubiquitylating enzymes, Cancer metabolism, Aerobic glycolysis, Fatty acid metabolism, Targeted therapy

## Abstract

Cancer cells must rewire cellular metabolism to satisfy the unbridled proliferation, and metabolic reprogramming provides not only the advantage for cancer cell proliferation but also new targets for cancer treatment. However, the plasticity of the metabolic pathways makes them very difficult to target. Deubiquitylating enzymes (DUBs) are proteases that cleave ubiquitin from the substrate proteins and process ubiquitin precursors. While the molecular mechanisms are not fully understood, many DUBs have been shown to be involved in tumorigenesis and progression via controlling the dysregulated cancer metabolism, and consequently recognized as potential drug targets for cancer treatment. In this article, we summarized the significant progress in understanding the key roles of DUBs in cancer cell metabolic rewiring and the opportunities for the application of DUBs inhibitors in cancer treatment, intending to provide potential implications for both research purpose and clinical applications.

**Supplementary Information:**

The online version contains supplementary material available at 10.1186/s12935-022-02524-y.

## Introduction

Tumorigenesis is dependent on the reprogramming of cellular metabolism, which has been recognized as one of the hallmarks of cancer [1, 2]. Cell proliferation requires nutrients, energy, and biosynthetic activities to duplicate all macromolecular components during each passage through the cell cycle. It is therefore not surprising that metabolic activities in uncontrolled cancer cells are fundamentally different from those in normal cells.

Interestingly, the dysregulated cancer cell metabolism provides not only proliferation advantages but also new targets for cancer diagnosis and therapy [3–6]. For instance, the enhanced glucose uptake by cancer cells allows the clinicians to image cancer using the glucose analog 2-(18F)-fluoro-2-deoxy-D-glucose (FDG) by positron emission tomography (PET) [7]. The FDG-PET combined with computer tomography (PET/CT) has a > 90% sensitivity and specificity for detection of metastases of most epithelial cancers [7]. Moreover, inhibitors of nucleotide metabolism (also known as antimetabolites), including methotrexate, 5-fluorouracil, 6-mercaptopurine and pemetrexed, which antagonize the activity of enzymes involved in nucleotide biosynthesis, have been successfully used in modern chemotherapy regimens to prolong cancer patient survival [8, 9]. Unfortunately, these chemotherapies are not tumor-specific, and frequently cause severe side effects due to on-target inhibition of the same enzymes in normal cells [10]. One exception is the recent success in the development of inhibitors targeting oncogenic isocitrate dehydrogenase 1 (IDH1) and IDH2 mutations. However, IDH-activating mutations, which were primarily identified in a subset of astrocytomas, oligodendrogliomas, gliomas and acute myeloid leukemias, are less frequently occurred in other human cancers [11, 12]. For other cancer types, it remains less clear which pathways of the cellular metabolism could represent a realistic, targetable vulnerability of tumor cells in comparison with normal counterparts. A better understanding of the underlying tumor-specific metabolic regulatory mechanisms may help develop and optimize novel therapeutic strategies targeting cancer cells [9, 10, 13].

While the detailed molecular mechanisms responsible for the abnormal cancer metabolism remain largely unknown, increasing number of studies have shown that deubiquitylating enzymes (DUBs) play a key role in governing tumor cell metabolic rewiring, including aerobic glycolysis, gluconeogenesis, de novo lipid synthesis, glutamine metabolism, and non-essential amino acid metabolism. In this review article, we aim to discuss the regulation of cancer metabolism by DUBs in carcinogenesis and the potential of targeting DUBs as strategies to improve cancer therapy.

### DUBs

**Fig. 1:**
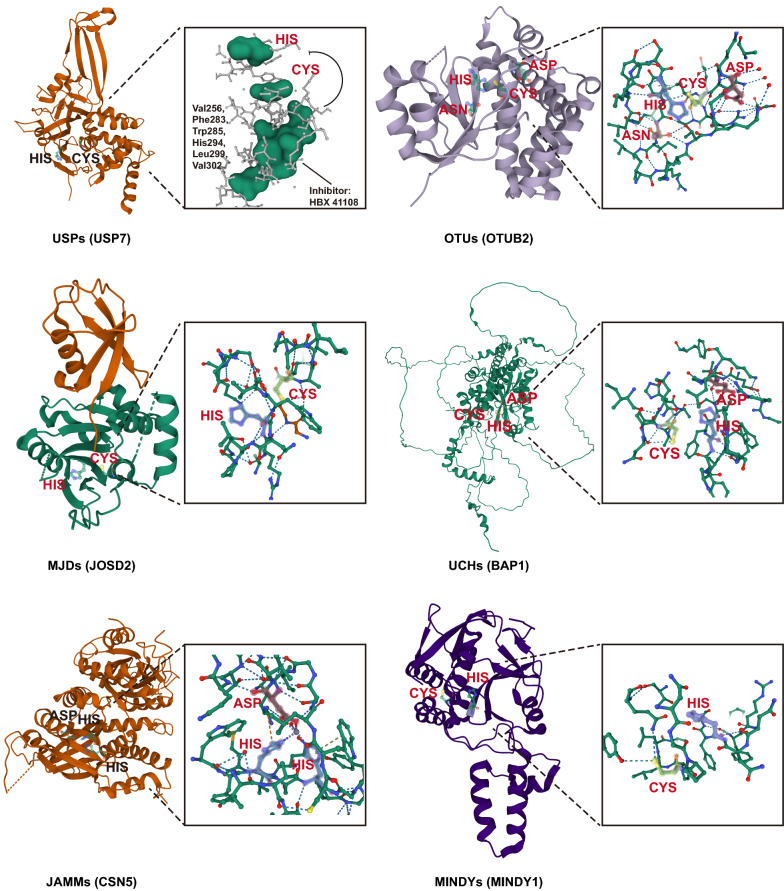
Structure of six representative subclasses of DUBs. The secondary structure is significantly different among these DUB classes, and the key catalytic site domains in each DUB are shown on the right of the structure. For USP7, we show a schematic diagram of the binding site and mechanism of action of one of the inhibitors. The labeled catalytic site information comes from UniProt (https://www.uniprot.org). UniProtKB and Protein Databank (PDB) codes: Ubiquitin specific peptidase 7 (USP7), Q93009, 1NB8; OTU deubiquitinase (ubiquitin aldehyde binding 2, OTUB2), Q96DC9, 1TFF; BRCA1 associated protein 1 (BAP1), Q92560, 1TQN; Josephin domain containing 2 (JOSD2), Q8TAC2, 6PGV; COP9 signalosome subunit 5 (CSN5), Q92905, 4F7O; MINDY lysine 48 deubiquitinase 1 (MINDY1), Q8N5J2, 5JKN (A chain)

The post-translational modification of cellular proteins through ubiquitylation is a dynamic and reversible process coordinated by the action of ubiquitin-conjugating enzymes and DUBs [14]. DUBs can remove ubiquitin chains or mono ubiquitin from post-translationally modified proteins, which not only can lead to protein stabilization by rescue from either proteasomal or lysosomal degradation, but also affect protein functioning by altering interactome and/or subcellular localization [15]. Moreover, DUBs are required for both generation and recycling of free ubiquitin, and therefore play a key role in maintaining the cellular ubiquitin homeostasis [15, 16]. Approximately 100 DUBs have been identified in the human genome, which can be categorized into six major subfamilies based on the active site homology. There are four families of Cys-dependent proteases, which contain a catalytic triad of Cys, His and Asp/Asn. Ubiquitin-specific proteases (USPs, 56 members) represent the bulk of the DUBs; Ovarian tumor proteases (OTUs,14 members) can be divided into three subclasses including otubains, OTUs and A20-like OTUs; Ubiquitin C-terminal hydrolases (UCHs) family was the first to be structurally characterized; Josephins (also termed MJDs) family contains a poly-Gln stretch, the extension of which leads to the neurodegenerative disorder Machado–Joseph disease (MJD) [16, 17]. Jad1/Pad/MPN-domain-containing metalloenzymes (JAMMs), containing zinc-dependent metalloproteases, are commonly found in association with large protein complexes [16, 17]. Motif interacting with ubiquitin-containing novel DUB family (MINDYs), a recently identified subfamily, is highly selective at cleaving K48-linked polyubiquitin [18] (Fig. 1).

A comprehensive analysis of human cancers by in situ hybridization indicated that DUBs are frequently dysregulated in tumor samples [19]. Indeed, plenty of DUBs were found to be highly expressed in tumor samples (Additional file 1: Table S1) and function as biomarkers for cancers [20]. The dysregulated DUBs have been shown to be involved in tumorigenesis via regulating the stability of specific oncoprotein or tumor suppressor substrates [16]. Moreover, the aberrantly expressed DUBs were proposed as potential therapeutic targets for cancer treatment because they may modulate protein fate in a cancer-specific manner [16, 17, 20–24].

### DUBs and aerobic glycolysis

**Fig. 2:**
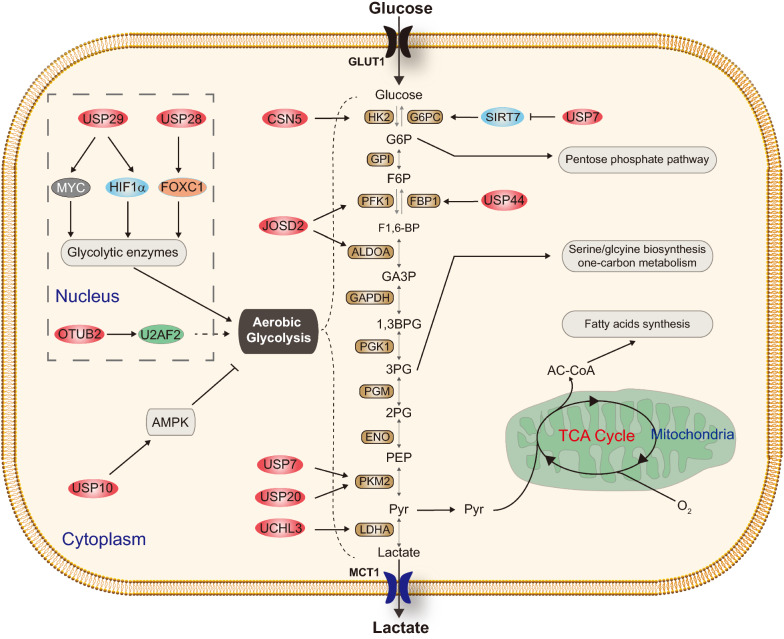
DUBs involved in the regulation of glucose metabolism. This figure shows that CSN5, JOSD2, OTUB2, USP7, USP10, USP20, USP29, and USP44 are involved in the regulation of glucose metabolism, respectively. *GPI* glucose 6-phosphate isomerase, *PGK1* phosphoglycerate kinase 1, *PGM* phosphoglucomutase, *ENO* α-Enolase, *LDHA* lactate dehydrogenase A, *Pyr* pyruvate, *AC-CoA* acetyl-CoA

In mammalian cells, glucose is one of the major sources of cellular energy and new cell mass. Glucose is metabolized via glycolysis to pyruvate, which can be oxidatively metabolized to CO_2_ in the tricarboxylic acid (TCA) cycle to generate a large amount of ATP through the process of oxidative phosphorylation (Fig. 2). Pyruvate can also be reductively metabolized to lactate, a process known as fermentation, which does not require oxygen but is far less efficient in ATP generation [25]. Tumor cells typically convert a majority of glucose to lactate even in the presence of oxygen, a phenomenon known as aerobic glycolysis or Warburg effect, which has been confirmed in a variety of tumor contexts and shown to correlate with poor prognosis [26]. The major function of aerobic glycolysis is to maintain high levels of glycolytic intermediates to support anabolic reactions in tumor cells [25, 27, 28].

Several DUBs were reported to be involved in aerobic glycolysis via regulating glycolytic enzymes. In non-small cell lung cancer (NSCLC), deubiquitinase Josephin Domain-containing protein 2 (JOSD2) was recently identified to display comprehensive effects on glucose catabolism, and thereby promoting cancer cell proliferation [29]. Mechanistically, JOSD2 stabilizes metabolic enzymes aldolase A (ALDOA) and phosphofructokinase-1 (PFK1) in vitro and in vivo. Furthermore, JOSD2 expression, but not a catalytically inactive mutant, deubiquitinates and stabilizes the enzyme complex, thereby enhancing their activities and the glycolytic rate [29]. In hepatocellular carcinoma (HCC) cells, depletion of CSN5 (also known as COP9 signalosome subunit 5, *COPS5*) caused a significant decrease in glucose uptake and the production of glycolytic intermediates. Mechanistically, CSN5 attenuated the ubiquitin–proteasome system-mediated degradation of hexokinase 2 (HK2) through its deubiquitinase function; resumption of HK2 expression rescued the decreased glycolytic flux induced by CSN5 knockdown, whereas inhibition of HK2 alleviated CSN5-enhanced glycolysis. Moreover, there was a positive correlation between CSN5 and HK2 in HCC samples [30]. Similarly, USP7 and USP20 were reported to deubiquitinate and stabilize pyruvate kinase isoenzyme M2 (PKM2) in Hela cells, indicating their roles in regulating glucose catabolism [31, 32] (Fig. 2).

DUBs are also involved in aerobic glycolysis via regulating transcription factors or signaling pathways. In our recent study, USP29 was identified to promote glucose consumption and lactate secretion in multiple cancer cells during both normoxia and hypoxia [33]. USP29 stabilizes oncogenic MYC (including c-MYC and N-MYC) and hypoxia-induced factor 1α (HIF1α), which are two major drivers of cancer metabolism in normoxia and hypoxia, respectively, by direct interaction and deubiquitination. Moreover, systematic knockout of *Usp29* in MYC-driven animal models markedly decreased the expression of intratumoral MYC, HIF1α, and their key downstream metabolic targets [33]. Consistently, another group recently reported that USP29 promotes aerobic glycolysis via stabilizing HIF1α to mediate sorafenib resistance in HCC cell lines, suggesting that USP29 may play a key role in the regulation of aerobic glycolysis in different cancer types [34]. In NSCLC, OTUB2 (OTU deubiquitinase, ubiquitin aldehyde binding 2) was significantly upregulated in primary tissues and associated with tumor malignancy [35]. Additional investigations showed that OTUB2 stabilizes U2 small nuclear RNA auxiliary factor 2 (U2AF2) to promote the Warburg effect and tumorigenesis via the AKT/mTOR signaling pathway [35]. In pancreatic cancer, over-expressed ubiquitin carboxyl-terminal hydrolase L3 (UCHL3) was reported to stabilize Forkhead box protein M1 (FOXM1), which activates the transcription of LDHA, and promotes aerobic glycolysis of pancreatic cancer through the UCHL3-FOXM1-LDHA axis [36]. In colorectal cancer (CRC) cells, knockdown of USP10 resulted in a significant increase in lactate production and glycolytic gene expression. USP10 specifically removes ubiquitination on the AMP-activated protein kinase (AMPK), which is a crucial sensor of the cellular response to low energy [37, 38]. On the other hand, USP10 is phosphorylated and activated by AMPK under energy stress. Thus the USP10-AMPK axis forms a positive feedforward loop to facilitate AMPK activation under energy stress [37, 38]. Although the detailed mechanisms remain unclear, USP28 was also reported to promote aerobic glycolysis of colorectal cancer by increasing stability of Forkhead Box C1 (FOXC1) [39] (Fig. 2).

### DUBs and gluconeogenesis

Gluconeogenesis is the synthesis of glucose from small carbohydrate precursors, such as lactate and amino acids [40]. The gluconeogenesis pathway is usually inhibited in cancers because it antagonizes glycolysis. However, some types of cancers rely on abbreviated forms of gluconeogenesis to support their bioenergetic and anabolic demands, especially under low glucose conditions; and thus, gluconeogenesis exerts context-dependent and highly important functions in tumorigenesis [40, 41]. The entire pathway of gluconeogenesis consists of eleven enzyme-catalyzed reactions, three of which are catalyzed exclusively by gluconeogenesis enzymes phosphoenolpyruvate carboxykinase (PEPCK), fructose-1,6-bisphosphatase (FBPase) and glucose-6-phosphatase (G6Pase) [40, 41].

In addition to aerobic glycolysis, DUBs also play important roles in cancer cell gluconeogenesis. Ecotopic expression of USP44 was reported to suppress the progression and overcome gemcitabine resistance of pancreatic ductal adenocarcinoma (PDAC) by suppressing glycolysis [42]. Further studies revealed that USP44 directly interacts with and stabilizes Fructose-1,6-bisphosphatase (FBP1), one of the key enzymes in the process of gluconeogenesis [42]. In CRC cells, USP7 was also reported to regulate gluconeogenesis through interacting with sirtuin 7 (SIRT7) and suppressing its enzymatic activity. SIRT7 is essential to the expression of glucose-6-phosphatase catalytic subunit (G6PC), a gluconeogenic gene [43] (Fig. 2).

### DUBs and fatty acid metabolism

Alterations in fatty acid metabolism in cancer cells are increasingly being recognized. Fatty acids (FAs) consist of a terminal carboxyl group and a hydrocarbon chain, mostly occurring in even numbers of carbons, that can be either saturated or unsaturated [44]. FAs are required for energy storage, membrane proliferation, and the generation of signaling molecules [44]. The cellular FAs come from either exogenous sources or de novo synthesis. Normal cells take up much of their required FAs from the circulation via the activity of lipoprotein lipase (LPL) and fatty acid translocases such as CD36 [45]. In contrast, cancer cells acquire their FAs mainly from the de novo fatty acid synthesis [46].

**Fig. 3:**
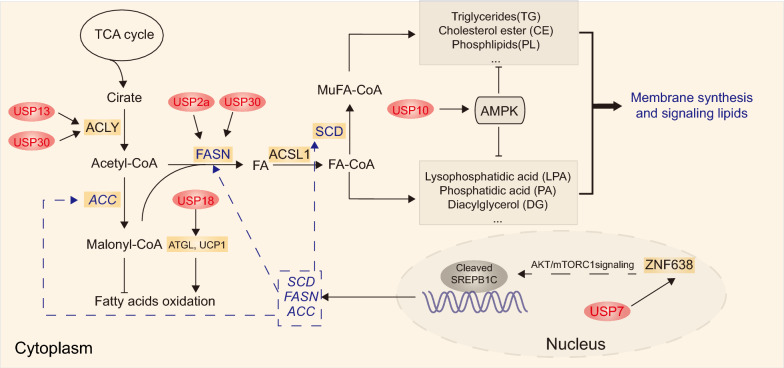
DUBs involved in the regulation of fatty acid metabolism. This figure shows that USP2a, USP7, USP10, USP13, USP18 and USP30 are involved in the regulation of fatty acid metabolism, respectively. *FA* fatty acid, *ACSL1* long-chain fatty acyl-CoA 1, *ZNF638* Zinc Finger Protein 638

Two key enzymes involved in de novo fatty acid synthesis were regulated by DUBs. In ovarian cancer, USP13 was shown to promote glutamine-dependent reductive carboxylation for lipogenesis [47]. Further investigation revealed that USP13 directly deubiquitinates and stabilizes ATP citrate lyase (ACLY), which is an important enzyme linking carbohydrate to lipid metabolism by generating acetyl-CoA from citrate for fatty acid and cholesterol biosynthesis [47]. In HCCs that arise in mice maintained on high-fat diets, USP30 was phosphorylated and stabilized by IKKβ, and USP30 deletion attenuated lipogenesis and tumorigenesis in DEN/CCl4-induced animal model [48]. The upregulated USP30 interacted with and stabilzed ACLY and fatty acid synthase (FASN) [49]. Moreover, USP2a was suggested to play a critical role in prostate cancer cell survival by deubiquitinating and stabilizing FASN [48] (Fig. 3).

DUBs also participate in de novo lipid synthesis via the regulation of abnormal signaling pathways. The Sterol Regulatory Element Binding Proteins (SREBPs), which include three isoforms (SREBP1a, SREBP1c and SREBP2), are the master regulators of lipid homeostasis, and SREBP-1c is the main transcription factor that mediates the activation of lipogenesis [50, 51]. USP7 was involved in the progression of lipogenesis-associated HCC by interacting with and stabilizing ZNF638, which may selectively increase the cleavage of SREBP-1c through AKT/mTORC1/S6K signaling pathway. The cleaved SREBP1c may transcriptionally activate lipogenesis-associated enzymes, including acetyl-CoA carboxylase (ACC), FASN, and Stearoyl-CoA desaturase (SCD) [52]. USP10 was also reported to suppress lipid synthesis by forming a positive feedback loop with APMK under energy stress in CRC cells [38] (Fig. 3).

In addition to de novo fatty acid synthesis, DUBs are also involved in fatty acid oxidation. Elevated levels of USP18 was reported to promote lipolysis, increase fatty acid oxidation and augment lung cancer growth; further investigation showed that USP18 directly stabilized adipose triglyceride lipase (ATGL) protein by removing Interferon-Stimulated Gene 15 (ISG15) from the conjugated complex, and stabilized Uncoupling Protein 1 (UCP1) via deubiquitination [53] (Fig. 3).

### DUBs and glutamine metabolism

Glutamine, which is the most abundant amino acid in blood, belongs to a group of conditionally essential amino acids [54, 55]. Many cancer cells exhibit an increased dependence on exogenous glutamine and become glutamine addicted [56]. Owing to glucose-derived pyruvate is mainly converted to lactate, glutamine is required for tumor cells to replenish the truncated TCA cycle through a process termed “anapleurosis” [28, 57–59]. Moreover, glutamine metabolism maintains mitochondrial integrity and NADPH levels needed for redox homeostasis and macromolecular synthesis [28, 57–59].

**Fig. 4:**
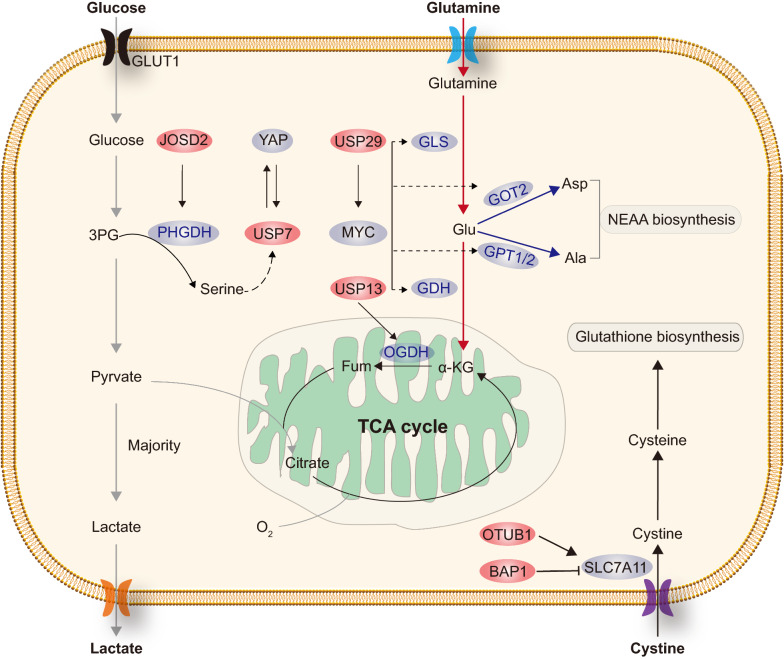
DUBs involved in the regulation of amino acids metabolism. This figure shows that BAP1, JOSD2, OTUB1, USP7, USP13 and USP29 are involved in the regulation of amino acids metabolism, respectively. *GLS* glutaminase, *GDH* glutamate dehydrogenase, *Asp* aspartate, *Ala* alanine

In human ovarian cancers, *USP13* was frequently amplified and showed to be critical for glutamine catobolism, and its depletion represses mitochondrial function [47]. USP13 may specifically deubiquitinates and thus upregulates oxoglutarate dehydrogenase (OGDH), a key enzyme that oxidizes α-KG to succinate [47]. In our recent study, USP29 played a key role in controling glutaminolysis in Burkitt’s lymphoma and Neuroblastoma [32]. USP29 deubiquitinates and stabilizes oncogenic MYC (including c-MYC and N-MYC), which directly activates the transcription of multiple genes involved in glutamine metabolism, including glutamate dehydrogenase 1(GLUD1), glutamic-oxaloacetic transaminase 2 (GOT2) and glutamic–pyruvic transaminase1/2 (GPT1/GPT2). These findings indicated that DUBs may play an important role in glutamine metabolism (Fig. 4).

### DUBs and metabolism of non-essential amino acids (NEAAs)

In addition to glutamine metabolism, accumulating evidence suggested that other non-essential amino acids (NEAAs) may also play critical roles in the pathogenesis of cancer [60]. NEAAs may influence tumor progression through macromolecule biosynthesis, maintenance of redox homeostasis, and numerous allosteric and epigenetic regulatory mechanisms [60].

Serine is involved in many crucial cellular processes, such as nucleotide synthesis, folate metabolism, and macromolecule synthesis [61]. Highly proliferative tumor cells exhibit strong demand for serine, which can be satisfied by enhancing either import from the extracellular environment or de novo synthesis from glucose. Notably, enhancement of the serine synthesis pathway (SSP) is a major metabolic reprogramming event that is important for oncogenic transformation in many cancers [62–65]. In NSCLC, JOSD2 was also identified as a positive regulator of SSP via deubiquitinating and stabilizing phosphoglycerate dehydrogenase (PHGDH), a key enzyme that drives the first committed step in de novo serine biosynthesis [29]. In colorectal cancer (CRC), USP7 was reported to promote serine deprivation resistance. Low concentration of cellular serine was found to suppress the expression of USP7 through an unknown mechanism [66]. USP7 deubiquitinates and stabilizes Yes-associated protein (YAP), which activates downstream signaling pathways and promotes cell proliferation [66] (Fig. 4).

Solute carrier family 7 member 11 (SLC7A11, also called XCT), the catalytic subunit of the cystine/glutamate amino acid transport system Xc-, is the major transporter of extracellular cystine [67–69]. Intracellular cystine is rapidly converted to cysteine, which subsequently serves as the rate-limiting precursor for glutathione synthesis [67–69]. Cystine depletion or drugs that block SLC7A11-mediated cystine uptake increase reactive oxygen species (ROS) and induce ferroptosis, which is an iron-dependent form of nonapoptotic cell death [70, 71]. BRCA1-associated protein 1 (BAP1) is a tumor suppressor gene with frequent inactivating mutations and deletions in human cancers [72]. Wildtype BAP1 was shown to inhibit cystine uptake, leading to ferroptosis and tumor suppression [73]. The mechanistic studies revealed that BAP1 reduced histone 2A ubiquitination (H2Aub) on the *SLC7A11* promoter and repressed SLC7A11 expression in a DUB-dependent manner [73]. OTUB1 (OTU deubiquitinase, ubiquitin aldehyde binding 1), which is overexpressed in a variety of human cancers, was shown to function as a major regulator for SLC7A11 stability [74]. OTUB1 interacted with and stabilized SLC7A11 in an enzyme activity-dependent manner. Functionally, the OTUB1-SLC7A11 axis was critical for tumor growth and OTUB1 inactivation promotes ferroptosis in human cancer cells primarily by down-regulating SLC7A11 levels [74] (Fig. 4).

### Targeting DUBs for cancer therapy

Targeting the dysregulated cancer metabolism has been recognized as a promising strategy for cancer treatment and multiple inhibitors directly targeting key metabolic enzymes have been developed [6]. In principle, direct inhibition of wild-type metabolic enzymes could cause severe “on-target” toxicity to normal tissues, since normal cells also depend on the same metabolic machinery. However, given the fact that many DUBs were highly elevated in various cancers and considered as cancer biomarkers [20], it is conceivable that targeting the key upstream regulators of metabolic enzymes, such as DUBs, may become an alternative approach for cancer therapy. The clinical application of lenalidomide (a ligand of ubiquitin E3 ligase cereblon) and bortezomib (targeting proteasome) in the treatment of multiple myeloma has facilitated the development of small-molecule inhibitors targeting other components of the ubiquitin proteasome system (UPS) [75]. Compared to E1 (Ub-activating enzymes) and E2 (Ub-conjugating), E3 ubiquitin ligases are more suitable targets for small-molecule inhibitors due to specificity concerns [76]. Interestingly, most DUBs are cysteine enzymes, which are ideal targets for the development of small molecule inhibitors [77], and are likely to be more druggable than E3 ligases owing to the lack of defined catalytic residues in the latter [17]. Indeed, dozens of DUB inhibitors have shown promising results in the preclinical studies (Table 1).

**Table1:** DUB inhibitors developed for cancer treatment

DUB	Target	Inhibitor	Functions affected by the inhibitor	Cancer type	Stage	Refs.
USP1	PCNA and FANCD2	Pimozide	Synthetic lethal with cisplatin	NSCLC	Preclinical	[111]
	PCNA and FANCD2	GW7647	Synthetic lethal with cisplatin	NSCLC	Preclinical	[111]
	ID1	C527	Growth inhibition	multiple myeloma	Preclinical	[112]
	ID proteins	SJB3-019A	Inhibition of DNA Repair and triggering apoptosis	multiple myeloma and B-ALL	Preclinical	[85, 86]
	PCNA,FANCD2 and KPNA2	ML323	DNA damage and suppression of metastasis	Osteosarcoma, NSCLC and breast cancer	Preclinical	[79, 83]
USP2	cyclin D1	ML364	Cell cycle arrest	CRC and Mantle Cell Lymphoma	Preclinical	[90]
	cyclin D1	LCAHA	G0/G1 arrest	CRC	Preclinical	[89]
	FASN, MDM2, cyclin D1 and Aurora-A	6TG	Cell killing and drug resistance	BRCA2 defective tumours	Preclinical	[113–115]
USP7	MDM2	HBX41108	Inhibition of Cell Proliferation	CRC	Preclinical	[116]
	SYK	HBX19818	Cell death	AML	Preclinical	[117]
	PLK1,Maf and N-MYC	P5091	Cell cycle and cell death	Multiple cancers	Preclinical	[94, 118–120]
	MDM2	GNE6640/6776	Synergy with PIM kinase inhibition	Breast cancer and Osteosarcoma	Preclinical	[121]
	MDM2	FT671/827	Inhibition of tumor growth	Osteosarcoma and CRC	Preclinical	[122]
	MDM2/MDM4	XL188	Cell death	Ewing sarcoma	Preclinical	[123, 124]
USP7/USP47	N-MYC, YB-1 et al	P22077	Drug resistance	Multiple cancers	Preclinical	[99–101, 125–128]
USP9X	Not reported	Degrasyn	Gemcitabine resistance	Pancreatic cancer	Preclinical	[129]
USP14	AR proteins	IU1	Inhibition of proliferation	Breast cancer	Preclinical	[130]
USP14/UCHL5	CXCR4	VLX1570	ER Stress	Multiple myeloma, ALL	Preclinical	[104, 106, 131]
	Proteasome	Auranofin	Apoptosis	Multiple cancers	Phase II	[108, 109]
UCHL1	TβRI and SMAD2	6RK73	Inhibition of migration and extravasation	Breast cancer	Preclinical	[132]
CSN5	Cullin-RING E3 ubiquitin ligases	CSN5i-3	Inhibition of tumor growth	Large cell lymphoma and CRC	Preclinical	[133]
RPN11	Proteins at the 19S proteasome entry gate	O-phenanthroline	Apoptosis	Multiple myeloma	Preclinical	[134]
	Proteins at the 19S proteasome entry gate	Quinoline-8-thiol	ER stress	CRC	Preclinical	[110]
Pan DUBs	Global protein stability	PR-619	ER stress, G2/M cell cycle arrest and apoptosis	Multiple cancers	Preclinical	[126, 135, 136]

## USP1 inhibitors

USP1 was reported to play an oncogenic role in multiple cancers via diverse mechanisms [78–80]. USP1 is associated with UAF1 (WDR48, also named USP1 associated factor 1) in tumor cells, and this interaction is important for its cellular function. In prostate cancer, USP1 was reported to stabilize histone demethylase lysine-specific demethylase 4A (KDM4A) and indirectly activates the expression of C-MYC, which is a driver of deregulated cancer metabolism; inhibition of USP1 by ML323, a nanomolar inhibitor of USP1-UAF1 with remarkable selectivity, caused a dramatic downregulation of C-MYC and sensitized cells to enzalutamide treatment [81, 82]. Moreover, ML323 was reported to potentiate cisplatin cytotoxicity in NSCLC and osteosarcoma cells [83], represses breast cancer metastasis [79] and promote CRC chemosensitivity [84]. SJB3-019A is an inhibitor that selectively blocks USP1 enzymatic activity, and treatment of multiple myeloma (MM) cells with SJB3-019A triggers apoptosis and downregulates MM stem cell renewal [85]. Similarly, SJB3-019A was also reported to repress cell proliferation and induce B-ALL cell apoptosis [86].

## USP2 inhibitors

USP2 was responsible for stabilizing many tumor-associated proteins, including FASN [48], mouse double minute 4 (MDM4)/MDMX [87, 88] and cyclin D1 [89]. ML364 is a small molucule inhibitor that directly binds to USP2. Ml364 was reported to induce cell cycle arrest in CRC and Mantle Cell Lymphoma [90] and dampen TGF-β-triggered signaling and metastasis in HCC [91]. In breast cancers, ML364 potentiates the pro-degradation effects of HSP90 inhibitors on ErbB2 and hence sensitizes ErbB2-positive cells to HSP90 inhibition. The combination of USP2 and HSP90 inhibitors effectively restrains ErbB2-positive breast cancer xenograft growth in vivo [92]. Lithocholic acid (LCA) derivatives were reported to function as USP inhibitors; lithocholic acid hydroxyamide (LCAHA), which is the most potent LCA derivative, was showed to inhibit USP2a (one isform of USP2) and arrest cell cycle [89].

## USP7 inhibitors

USP7 plays comprehensive roles in cancers by regulating both oncogenic drivers and tumor suppressors, such as N-MYC, HIF1α, Notch Receptor 1 (Notch1), MDM2, p53, and Phosphatase and Tensin Homolog (PTEN) [93]. Several small molecule inhibitors of USP7 have been developed for cancer treatment, of which P5091 and P22077 were most widely studied. P5091, a tri-substituted thiophene with dichlorophenylthio, nitro, and acetyl substituents mediating anti-USP7 activity, was firstly reported to induce apoptosis in multiple myeloma cells resistant to conventional and bortezomib therapies [94], and then showed antitumor effect in various cancers, including CRC, ovarian cancer, bladder cancer, prostate cancer and HCC [95, 96]. In gastric cancer, depletion of USP7 by p5091 decreased PD-L1 (Programmed Cell Death 1 Ligand 1, also known as CD274) expression and sensitized gastric cancer cells to T cell killing [97]. Moreover, p5091 was reported to significantly modulate the phenotype and function of M2 (CD11b^+^F4/80^+^CD86^−^CD206^+^) macrophages, and combinational treatment of p5091 and Programmed Cell Death 1 (PD1) antibody exerted synergistic anti-tumor effect in lung cancer [98]. These studies suggest that combinational therapy with specific DUB inhibitors and immune checkpoint inhibitors (e.g. PD-1/PD-L1 antibodies) may become another innovative approach for cancer treatment in future. P22077, a selective inhibitor of USP7 and the related protein USP47, was shown to induce cytotoxicity in chronic myelogenous leukemia (CML) cells with or without TKI resistance and eliminates leukemia stem/progenitor cells in CML mice [85, 99]. P22077 was also found to be able to overcome chemoresistance in *MYCN*-amplified neuroblastoma, HCC and pancreatic cancer [87, 100, 101].

## Inhibitors for proteasome-associated DUBs

USP14, UCHL5 (Ubiquitin C-Terminal Hydrolase L5) and Rpn11 (Proteasome 26S Subunit, Non-ATPase 14, PSMD14, also known as Rpn11) are three proteasome-associated DUBs. While Rpn11 is an integral part of the proteasome, association of USP14 and UCLH5 with the 19S RP is transient [102]. Inhibition of proteasome deubiquitinating activity is relatively a new cancer therapy strategy [103]. VLX1570, a dual USP14/UCHL5 inhibitor, was reported to induce apoptosis of multiple myeloma cells [104]. VLX1570 was approved to undergo phase I clinical trial in patients with confirmed diagnosis of multiple myeloma with relapsed and/or refractory disease, but the clinical trial was suspended due to dose-limiting toxicity [105]. Interestingly, VLX1570 was also showed to induce cytotoxicity in Acute Lymphoblastic Leukemia (ALL) [106, 107]. Auranofin, a gold-containing compound clinically used to treat rheumatic arthritis, was recently approved for Phase II clinical trial to treat chronic lymphocytic leukemia (CLL) but its anti-cancer mechanism is poorly understood. Auranofin was reported to induce cytotoxicity by targeting UCHL5/USP14 in various cancers [108, 109]. Rpn11 is a member of the JAMM zinc metalloprotease family of DUBs and a catalytic subunit of the 19S regulatory particle of the proteasome. Capzimin, which was developed and optimized based on the Rpn11 inhibitor Quinoline-8-thiol (1, 8TQ), causes a broad inhibition of cancer cell proliferation [110].

## Conclusion and perspective

**Fig. 5:**
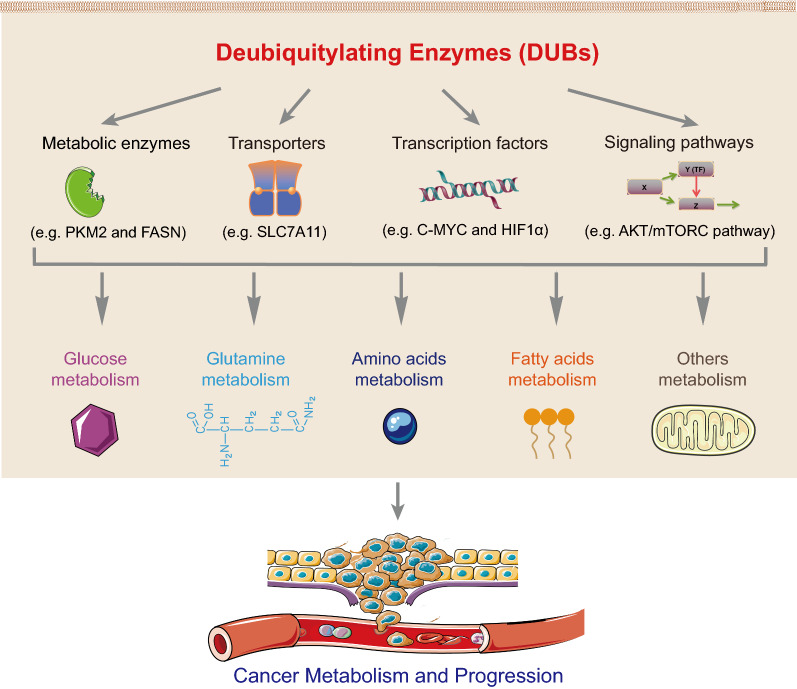
A schematic diagram illustrating the roles of DUBs in cancer metabolism and progression. DUBs are involved in multiple metabolic processes, including glucose, glutamine, amino acids and fatty acids metabolism through regulation of metabolic enzymes, transporters, transcription factors and signaling pathways, and play key roles in tumorigenesis and progression

DUBs have been shown to be involved in many aspects of metabolic processes, including glucose, glutamine, amino acids and fatty acids metabolism via regulating the metabolic enzymes, transporters, transcription factors and/or signaling pathways, and to play important roles in tumorigenesis and progression by modulating cancer cell metabolism (Fig. 5). Despite tremendous progress have been made in the past decade, many important questions remain to be addressed in order to have a better understanding of the comprehensive roles of DUBs in cancer metabolism. For instance, the upstream regulatory mechanisms of DUBs and whether the cancer cell metabolic rewiring affects the expression or activity of DUB remain largely unknown. Systemic knockout of many DUBs did not exhibit obvious effect on the growth and development in animal models, but significantly inhibited tumorigenesis (e.g. USP29 and USP30), indicating that they may specifically function in cancer. Thus, it is urgent to analyze their protein structures and develop highly selective small molecule inhibitors against cancer. These studies will not only help us to further characterize the DUBs associated with cancer metabolism, but also identify novel potent and cancer-specific DUB inhibitors for cancer target therapy. Moreover, it is conceivable that combinational therapy with specific DUB inhibitors and other types of target therapeutic agents (e.g. inhibitors targeting EGFR mutations) as well as immune checkpoints inhibitors (e.g. PD-1/PD-L1 antibodies) may become another innovative approach for cancer treatment in future.

## Supplementary Information

**Additional file 1: Table S1.** List of DUBs highly expressed in cancers.

## Data Availability

Not applicable.

## Abbreviations

AC-CoA: Acetyl-CoA
ACLY: ATP citrate lyase
ACSL1: Long-chain fatty acyl-CoA 1
Ala: Alanine
Asp: Aspartate
BAP1: BRCA1-associated protein 1
CSN5: COP9 signalosome subunit 5
DUBs: Deubiquitylating enzymes
ENO: α-Enolase
FA: Fatty acid
FASN: Fatty acid synthase
FDG: 2-(18F)-fluoro-2-deoxy-D-glucose
GDH: Glutamate dehydrogenase
GLS: Glutaminase
GPI: Glucose 6-phosphate isomerase
HCC: Hepatocellular carcinoma
HIF1α: Hypoxia-induced factor 1α
HK2: Hexokinase 2
IDH1: Isocitrate dehydrogenase 1
JAMMs: Josephins and Jad1/Pad/MPN-domain-containing metalloenzymes
LDHA: Lactate dehydrogenase A Lactate dehydrogenase A
LPL: Lipoprotein lipase
MINDYs: Motif interacting with ubiquitin-containing novel DUB family
NSCLC: Non-small cell lung cancer
OTUs: Ovarian tumor proteases
PFK1: Phosphofructokinase-1
PGK1: Phosphoglycerate kinase 1
PGM: Phosphoglucomutase
PKM2: Pyruvate kinase isoenzyme M2
Pyr: Pyruvate
SCD: Stearoyl-CoA desaturase
SIRT7: Sirtuin 7
SREBPs: Sterol Regulatory Element Binding Proteins
TCA: Tricarboxylic acid
TCGA: The cancer genome atlas
UCHs: Ubiquitin C-terminal hydrolases
USPs: Ubiquitin-specific proteases
ZNF638: Zinc Finger Protein 638

## References

[1] Hanahan D, Weinberg RA (2011). Hallmarks of cancer: the next generation. Cell.

[2] Faubert B, Solmonson A, DeBerardinis RJ (2020). Metabolic reprogramming and cancer progression. Science.

[3] DeBerardinis RJ, Lum JJ, Hatzivassiliou G, Thompson CB (2008). The biology of cancer: metabolic reprogramming fuels cell growth and proliferation. Cell Metab.

[4] Zhu J, Thompson CB (2019). Metabolic regulation of cell growth and proliferation. Nat Rev Mol Cell Biol.

[5] Kroemer G, Pouyssegur J (2008). Tumor cell metabolism: cancer's Achilles' heel. Cancer Cell.

[6] Vernieri C, Casola S, Foiani M, Pietrantonio F, de Braud F, Longo V (2016). Targeting cancer metabolism: dietary and pharmacologic interventions. Cancer Discov.

[7] Mankoff DA, Eary JF, Link JM, Muzi M, Rajendran JG, Spence AM, Krohn KA (2007). Tumor-specific positron emission tomography imaging in patients: [18F] fluorodeoxyglucose and beyond. Clin Cancer Res.

[8] Vander Heiden MG, DeBerardinis RJ (2017). Understanding the intersections between metabolism and cancer biology. Cell.

[9] Vander Heiden MG (2011). Targeting cancer metabolism: a therapeutic window opens. Nat Rev Drug Discov.

[10] Tennant DA, Duran RV, Gottlieb E (2010). Targeting metabolic transformation for cancer therapy. Nat Rev Cancer.

[11] Yan H, Parsons DW, Jin G, McLendon R, Rasheed BA, Yuan W, Kos I, Batinic-Haberle I, Jones S, Riggins GJ (2009). IDH1 and IDH2 mutations in gliomas. N Engl J Med.

[12] Mardis ER, Ding L, Dooling DJ, Larson DE, McLellan MD, Chen K, Koboldt DC, Fulton RS, Delehaunty KD, McGrath SD (2009). Recurring mutations found by sequencing an acute myeloid leukemia genome. N Engl J Med.

[13] Luengo A, Gui DY, Vander Heiden MG (2017). Targeting metabolism for cancer therapy. Cell Chem Biol.

[14] Fraile JM, Quesada V, Rodriguez D, Freije JM, Lopez-Otin C (2012). Deubiquitinases in cancer: new functions and therapeutic options. Oncogene.

[15] Komander D, Clague MJ, Urbe S (2009). Breaking the chains: structure and function of the deubiquitinases. Nat Rev Mol Cell Biol.

[16] Cheng J, Guo J, North BJ, Wang B, Cui CP, Li H, Tao K, Zhang L, Wei W (2019). Functional analysis of deubiquitylating enzymes in tumorigenesis and development. Biochimica Biophys Acta Rev Cancer.

[17] D'Arcy P, Wang X, Linder S (2015). Deubiquitinase inhibition as a cancer therapeutic strategy. Pharmacol Ther.

[18] Abdul Rehman SA, Kristariyanto YA, Choi SY, Nkosi PJ, Weidlich S, Labib K, Hofmann K, Kulathu Y (2016). MINDY-1 Is a member of an evolutionarily conserved and structurally distinct new family of deubiquitinating enzymes. Mol Cell.

[19] Luise C, Capra M, Donzelli M, Mazzarol G, Jodice MG, Nuciforo P, Viale G, Di Fiore PP, Confalonieri S (2011). An atlas of altered expression of deubiquitinating enzymes in human cancer. PLoS ONE.

[20] Poondla N, Chandrasekaran AP, Kim KS, Ramakrishna S (2019). Deubiquitinating enzymes as cancer biomarkers: new therapeutic opportunities?. BMB Rep.

[21] Singh N, Singh AB (2016). Deubiquitinases and cancer: A snapshot. Crit Rev Oncol Hematol.

[22] Harrigan JA, Jacq X, Martin NM, Jackson SP (2018). Deubiquitylating enzymes and drug discovery: emerging opportunities. Nat Rev Drug Discovery.

[23] Kaushal K, Antao AM, Kim KS, Ramakrishna S (2018). Deubiquitinating enzymes in cancer stem cells: functions and targeted inhibition for cancer therapy. Drug Discovery Today.

[24] Schauer NJ, Magin RS, Liu X, Doherty LM, Buhrlage SJ (2019). Advances in Discovering Deubiquitinating Enzyme (DUB) Inhibitors. J Med Chem.

[25] Lunt SY, Vander Heiden MG (2011). Aerobic glycolysis: meeting the metabolic requirements of cell proliferation. Annu Rev Cell Dev Biol.

[26] Warburg O (1956). On respiratory impairment in cancer cells. Science.

[27] Vander Heiden MG, Cantley LC, Thompson CB (2009). Understanding the Warburg effect: the metabolic requirements of cell proliferation. Science.

[28] Pavlova NN, Thompson CB (2016). The emerging hallmarks of cancer metabolism. Cell Metab.

[29] Krassikova L, Zhang B, Nagarajan D, Queiroz AL, Kacal M, Samakidis E, Vakifahmetoglu-Norberg H, Norberg E (2021). The deubiquitinase JOSD2 is a positive regulator of glucose metabolism. Cell Death Differ.

[30] Huang M, Xiong H, Luo D, Xu B, Liu H (2020). CSN5 upregulates glycolysis to promote hepatocellular carcinoma metastasis via stabilizing the HK2 protein. Exp Cell Res.

[31] Choi HS, Pei CZ, Park JH, Kim SY, Song SY, Shin GJ, Baek KH (2020). Protein stability of pyruvate kinase isozyme M2 is mediated by HAUSP. Cancers (Basel).

[32] Kim SR, Kim JO, Lim KH, Yun JH, Han I, Baek KH (2015). Regulation of pyruvate kinase isozyme M2 is mediated by the ubiquitin-specific protease 20. Int J Oncol.

[33] Tu R, Kang W, Yang M, Wang L, Bao Q, Chen Z, Dong Y, Wang J, Jiang J, Liu H (2021). USP29 coordinates MYC and HIF1alpha stabilization to promote tumor metabolism and progression. Oncogene.

[34] Gao R, Buechel D, Kalathur RKR, Morini MF, Coto-Llerena M, Ercan C, Piscuoglio S, Chen Q, Blumer T, Wang X (2021). USP29-mediated HIF1alpha stabilization is associated with Sorafenib resistance of hepatocellular carcinoma cells by upregulating glycolysis. Oncogenesis.

[35] Li J, Cheng D, Zhu M, Yu H, Pan Z, Liu L, Geng Q, Pan H, Yan M, Yao M (2019). OTUB2 stabilizes U2AF2 to promote the Warburg effect and tumorigenesis via the AKT/mTOR signaling pathway in non-small cell lung cancer. Theranostics.

[36] Fan Y, Hu D, Li D, Ma C, Tang Y, Tao Q, Deng L, Tang D (2021). UCHL3 promotes aerobic glycolysis of pancreatic cancer through upregulating LDHA expression. Clin Transl Oncol.

[37] Carling D, Mayer FV, Sanders MJ, Gamblin SJ (2011). AMP-activated protein kinase: nature's energy sensor. Nat Chem Biol.

[38] Deng M, Yang X, Qin B, Liu T, Zhang H, Guo W, Lee SB, Kim JJ, Yuan J, Pei H (2016). Deubiquitination and activation of AMPK by USP10. Mol Cell.

[39] Liu Z, Chen M, Xu X, Zhang L, Pan Y, Chen D (2021). USP28 promotes aerobic glycolysis of colorectal cancer by increasing stability of FOXC1. Acta Biochim Pol.

[40] Wang Z, Dong C (2019). Gluconeogenesis in Cancer: Function and Regulation of PEPCK, FBPase, and G6Pase. Trends Cancer.

[41] Grasmann G, Smolle E, Olschewski H, Leithner K (2019). Gluconeogenesis in cancer cells - Repurposing of a starvation-induced metabolic pathway?. Biochim Biophys Acta.

[42] Yang C, Zhu S, Yang H, Deng S, Fan P, Li M, Jin X (2019). USP44 suppresses pancreatic cancer progression and overcomes gemcitabine resistance by deubiquitinating FBP1. Am J Cancer Res.

[43] Jiang L, Xiong J, Zhan J, Yuan F, Tang M, Zhang C, Cao Z, Chen Y, Lu X, Li Y (2017). Ubiquitin-specific peptidase 7 (USP7)-mediated deubiquitination of the histone deacetylase SIRT7 regulates gluconeogenesis. J Biol Chem.

[44] Currie E, Schulze A, Zechner R, Walther TC, Farese RV (2013). Cellular fatty acid metabolism and cancer. Cell Metab.

[45] Goldberg IJ, Eckel RH, Abumrad NA (2009). Regulation of fatty acid uptake into tissues: lipoprotein lipase- and CD36-mediated pathways. J Lipid Res.

[46] Medes G, Thomas A, Weinhouse S (1953). Metabolism of neoplastic tissue. IV. A study of lipid synthesis in neoplastic tissue slices in vitro. Cancer Res.

[47] Han C, Yang L, Choi HH, Baddour J, Achreja A, Liu Y, Li Y, Li J, Wan G, Huang C (2016). Amplification of USP13 drives ovarian cancer metabolism. Nat Commun.

[48] Graner E, Tang D, Rossi S, Baron A, Migita T, Weinstein LJ, Lechpammer M, Huesken D, Zimmermann J, Signoretti S (2004). The isopeptidase USP2a regulates the stability of fatty acid synthase in prostate cancer. Cancer Cell.

[49] Gu L, Zhu Y, Lin X, Lu B, Zhou X, Zhou F, Zhao Q, Prochownik EV, Li Y (2021). The IKKbeta-USP30-ACLY Axis Controls Lipogenesis and Tumorigenesis. Hepatology.

[50] Eberle D, Hegarty B, Bossard P, Ferre P, Foufelle F (2004). SREBP transcription factors: master regulators of lipid homeostasis. Biochimie.

[51] Shimano H, Sato R (2017). SREBP-regulated lipid metabolism: convergent physiology - divergent pathophysiology. Nat Rev Endocrinol.

[52] Ni W, Lin S, Bian S, Zheng W, Qu L, Fan Y, Lu C, Xiao M, Zhou P (2020). USP7 mediates pathological hepatic de novo lipogenesis through promoting stabilization and transcription of ZNF638. Cell Death Dis.

[53] Liu X, Lu Y, Chen Z, Liu X, Hu W, Zheng L, Chen Y, Kurie JM, Shi M, Mustachio LM (2021). The ubiquitin-specific peptidase USP18 promotes lipolysis, fatty acid oxidation, and lung cancer growth. Mol Cancer Res.

[54] Bergstrom J, Furst P, Noree LO, Vinnars E (1974). Intracellular free amino acid concentration in human muscle tissue. J Appl Physiol.

[55] Lacey JM, Wilmore DW (1990). Is glutamine a conditionally essential amino acid?. Nutr Rev.

[56] Wise DR, Thompson CB (2010). Glutamine addiction: a new therapeutic target in cancer. Trends Biochem Sci.

[57] Wise DR, DeBerardinis RJ, Mancuso A, Sayed N, Zhang XY, Pfeiffer HK, Nissim I, Daikhin E, Yudkoff M, McMahon SB (2008). Myc regulates a transcriptional program that stimulates mitochondrial glutaminolysis and leads to glutamine addiction. Proc Natl Acad Sci USA.

[58] Qing G, Li B, Vu A, Skuli N, Walton ZE, Liu X, Mayes PA, Wise DR, Thompson CB, Maris JM (2012). ATF4 regulates MYC-mediated neuroblastoma cell death upon glutamine deprivation. Cancer Cell.

[59] Altman BJ, Stine ZE, Dang CV (2016). From Krebs to clinic: glutamine metabolism to cancer therapy. Nat Rev Cancer.

[60] Choi BH, Coloff JL (2019). The Diverse Functions of Non-Essential Amino Acids in Cancer. Cancers (Basel).

[61] Li AM, Ye J (2020). Reprogramming of serine, glycine and one-carbon metabolism in cancer. Biochim Biophys Acta Mol Basis Dis.

[62] Possemato R, Marks KM, Shaul YD, Pacold ME, Kim D, Birsoy K, Sethumadhavan S, Woo HK, Jang HG, Jha AK (2011). Functional genomics reveal that the serine synthesis pathway is essential in breast cancer. Nature.

[63] DeNicola GM, Chen PH, Mullarky E, Sudderth JA, Hu Z, Wu D, Tang H, Xie Y, Asara JM, Huffman KE (2015). NRF2 regulates serine biosynthesis in non-small cell lung cancer. Nat Genet.

[64] Sullivan MR, Mattaini KR, Dennstedt EA, Nguyen AA, Sivanand S, Reilly MF, Meeth K, Muir A, Darnell AM, Bosenberg MW (2019). Increased serine synthesis provides an advantage for tumors arising in tissues where serine levels are limiting. Cell Metab.

[65] Ngo B, Kim E, Osorio-Vasquez V, Doll S, Bustraan S, Liang RJ, Luengo A, Davidson SM, Ali A, Ferraro GB (2020). Limited environmental serine and glycine confer brain metastasis sensitivity to PHGDH Inhibition. Cancer Discov.

[66] Zhao X, Fu J, Hu B, Chen L, Wang J, Fang J, Ge C, Lin H, Pan K, Fu L (2021). Serine metabolism regulates YAP activity through USP7 in colon cancer. Front Cell Dev Biol.

[67] Lim JC, Donaldson PJ (2011). Focus on molecules: the cystine/glutamate exchanger (System x(c)(-)). Exp Eye Res.

[68] Conrad M, Sato H (2012). The oxidative stress-inducible cystine/glutamate antiporter, system x (c) (-): cystine supplier and beyond. Amino Acids.

[69] Koppula P, Zhang Y, Zhuang L, Gan B (2018). Amino acid transporter SLC7A11/xCT at the crossroads of regulating redox homeostasis and nutrient dependency of cancer. Cancer Commun (Lond).

[70] Dixon SJ, Lemberg KM, Lamprecht MR, Skouta R, Zaitsev EM, Gleason CE, Patel DN, Bauer AJ, Cantley AM, Yang WS (2012). Ferroptosis: an iron-dependent form of nonapoptotic cell death. Cell.

[71] Cramer SL, Saha A, Liu J, Tadi S, Tiziani S, Yan W, Triplett K, Lamb C, Alters SE, Rowlinson S (2017). Systemic depletion of L-cyst(e)ine with cyst(e)inase increases reactive oxygen species and suppresses tumor growth. Nat Med.

[72] Carbone M, Yang H, Pass HI, Krausz T, Testa JR, Gaudino G (2013). BAP1 and cancer. Nat Rev Cancer.

[73] Zhang Y, Shi J, Liu X, Feng L, Gong Z, Koppula P, Sirohi K, Li X, Wei Y, Lee H (2018). BAP1 links metabolic regulation of ferroptosis to tumour suppression. Nat Cell Biol.

[74] Liu T, Jiang L, Tavana O, Gu W (2019). The deubiquitylase OTUB1 mediates ferroptosis via stabilization of SLC7A11. Cancer Res.

[75] Shen M, Schmitt S, Buac D, Dou QP (2013). Targeting the ubiquitin-proteasome system for cancer therapy. Expert Opin Ther Targets.

[76] LaPlante G, Zhang W (2021). Targeting the ubiquitin-proteasome system for cancer therapeutics by small-molecule inhibitors. Cancers (Basel).

[77] Serafimova IM, Pufall MA, Krishnan S, Duda K, Cohen MS, Maglathlin RL, McFarland JM, Miller RM, Frodin M, Taunton J (2012). Reversible targeting of noncatalytic cysteines with chemically tuned electrophiles. Nat Chem Biol.

[78] Sonego M, Pellarin I, Costa A, Vinciguerra GLR, Coan M, Kraut A, D'Andrea S, Dall'Acqua A, Castillo-Tong DC, Califano D (2019). USP1 links platinum resistance to cancer cell dissemination by regulating Snail stability. Sci Adv.

[79] Ma A, Tang M, Zhang L, Wang B, Yang Z, Liu Y, Xu G, Wu L, Jing T, Xu X (2019). USP1 inhibition destabilizes KPNA2 and suppresses breast cancer metastasis. Oncogene.

[80] Ma L, Lin K, Chang G, Chen Y, Yue C, Guo Q, Zhang S, Jia Z, Huang TT, Zhou A (2019). Aberrant activation of beta-catenin signaling drives glioma tumorigenesis via USP1-mediated stabilization of EZH2. Can Res.

[81] Cui SZ, Lei ZY, Guan TP, Fan LL, Li YQ, Geng XY, Fu DX, Jiang HW, Xu SH (2020). Targeting USP1-dependent KDM4A protein stability as a potential prostate cancer therapy. Cancer Sci.

[82] Dong Y, Tu R, Liu H, Qing G (2020). Regulation of cancer cell metabolism: oncogenic MYC in the driver's seat. Signal Transduct Target Ther.

[83] Liang Q, Dexheimer TS, Zhang P, Rosenthal AS, Villamil MA, You C, Zhang Q, Chen J, Ott CA, Sun H (2014). A selective USP1-UAF1 inhibitor links deubiquitination to DNA damage responses. Nat Chem Biol.

[84] Xu X, Li S, Cui X, Han K, Wang J, Hou X, Cui L, He S, Xiao J, Yang Y (2019). Inhibition of ubiquitin specific protease 1 sensitizes colorectal cancer cells to DNA-damaging chemotherapeutics. Front Oncol.

[85] Das DS, Das A, Ray A, Song Y, Samur MK, Munshi NC, Chauhan D, Anderson KC (2017). Blockade of deubiquitylating enzyme USP1 inhibits DNA repair and triggers apoptosis in multiple myeloma cells. Clin Cancer Res.

[86] Kuang X, Xiong J, Lu T, Wang W, Zhang Z, Wang J (2021). Inhibition of USP1 induces apoptosis via ID1/AKT pathway in B-cell acute lymphoblastic leukemia cells. Int J Med Sci.

[87] Wang CL, Wang JY, Liu ZY, Ma XM, Wang XW, Jin H, Zhang XP, Fu D, Hou LJ, Lu YC (2014). Ubiquitin-specific protease 2a stabilizes MDM4 and facilitates the p53-mediated intrinsic apoptotic pathway in glioblastoma. Carcinogenesis.

[88] Allende-Vega N, Sparks A, Lane DP, Saville MK (2010). MdmX is a substrate for the deubiquitinating enzyme USP2a. Oncogene.

[89] Magiera K, Tomala M, Kubica K, De Cesare V, Trost M, Zieba BJ, Kachamakova-Trojanowska N, Les M, Dubin G, Holak TA (2017). Lithocholic Acid Hydroxyamide Destabilizes Cyclin D1 and Induces G0/G1 Arrest by Inhibiting Deubiquitinase USP2a. Cell Chem Biol.

[90] Davis MI, Pragani R, Fox JT, Shen M, Parmar K, Gaudiano EF, Liu L, Tanega C, McGee L, Hall MD (2016). Small molecule inhibition of the ubiquitin-specific protease USP2 Accelerates cyclin D1 degradation and leads to cell cycle arrest in colorectal cancer and mantle cell lymphoma models. J Biol Chem.

[91] Zhao Y, Wang X, Wang Q, Deng Y, Li K, Zhang M, Zhang Q, Zhou J, Wang HY, Bai P (2018). USP2a supports metastasis by tuning TGF-beta signaling. Cell Rep.

[92] Zhang J, Liu S, Li Q, Shi Y, Wu Y, Liu F, Wang S, Zaky MY, Yousuf W, Sun Q (2020). The deubiquitylase USP2 maintains ErbB2 abundance via counteracting endocytic degradation and represents a therapeutic target in ErbB2-positive breast cancer. Cell Death Differ.

[93] Song MS, Salmena L, Carracedo A, Egia A, Lo-Coco F, Teruya-Feldstein J, Pandolfi PP (2008). The deubiquitinylation and localization of PTEN are regulated by a HAUSP-PML network. Nature.

[94] Chauhan D, Tian Z, Nicholson B, Kumar KG, Zhou B, Carrasco R, McDermott JL, Leach CA, Fulcinniti M, Kodrasov MP (2012). A small molecule inhibitor of ubiquitin-specific protease-7 induces apoptosis in multiple myeloma cells and overcomes bortezomib resistance. Cancer Cell.

[95] An T, Gong Y, Li X, Kong L, Ma P, Gong L, Zhu H, Yu C, Liu J, Zhou H (2017). USP7 inhibitor P5091 inhibits Wnt signaling and colorectal tumor growth. Biochem Pharmacol.

[96] Ye M, He J, Zhang J, Liu B, Liu X, Xie L, Wei M, Dong R, Li K, Ma D (2021). USP7 promotes hepatoblastoma progression through activation of PI3K/AKT signaling pathway. Cancer Biomark.

[97] Wang Z, Kang W, Li O, Qi F, Wang J, You Y, He P, Suo Z, Zheng Y, Liu HM (2021). Abrogation of USP7 is an alternative strategy to downregulate PD-L1 and sensitize gastric cancer cells to T cells killing. Acta Pharm Sin B.

[98] Dai X, Lu L, Deng S, Meng J, Wan C, Huang J, Sun Y, Hu Y, Wu B, Wu G (2020). USP7 targeting modulates anti-tumor immune response by reprogramming Tumor-associated Macrophages in Lung Cancer. Theranostics.

[99] Lei H, Xu HZ, Shan HZ, Liu M, Lu Y, Fang ZX, Jin J, Jing B, Xiao XH, Gao SM (2021). Targeting USP47 overcomes tyrosine kinase inhibitor resistance and eradicates leukemia stem/progenitor cells in chronic myelogenous leukemia. Nat Commun.

[100] Zhang W, Zhang J, Xu C, Zhang S, Bian S, Jiang F, Ni W, Qu L, Lu C, Ni R (2020). Ubiquitin-specific protease 7 is a drug-able target that promotes hepatocellular carcinoma and chemoresistance. Cancer Cell Int.

[101] Chen H, Zhu X, Sun R, Ma P, Zhang E, Wang Z, Fan Y, Zhou G, Mao R (2020). Ubiquitin-specific protease 7 is a druggable target that is essential for pancreatic cancer growth and chemoresistance. Invest New Drugs.

[102] de Poot SAH, Tian G, Finley D (2017). Meddling with fate: the proteasomal deubiquitinating enzymes. J Mol Biol.

[103] D'Arcy P, Brnjic S, Olofsson MH, Fryknas M, Lindsten K, De Cesare M, Perego P, Sadeghi B, Hassan M, Larsson R (2011). Inhibition of proteasome deubiquitinating activity as a new cancer therapy. Nat Med.

[104] Wang X, Mazurkiewicz M, Hillert EK, Olofsson MH, Pierrou S, Hillertz P, Gullbo J, Selvaraju K, Paulus A, Akhtar S (2016). The proteasome deubiquitinase inhibitor VLX1570 shows selectivity for ubiquitin-specific protease-14 and induces apoptosis of multiple myeloma cells. Sci Rep.

[105] Rowinsky EK, Paner A, Berdeja JG, Paba-Prada C, Venugopal P, Porkka K, Gullbo J, Linder S, Loskog A, Richardson PG (2020). Phase 1 study of the protein deubiquitinase inhibitor VLX1570 in patients with relapsed and/or refractory multiple myeloma. Invest New Drugs.

[106] Pellegrini P, Selvaraju K, Faustini E, Mofers A, Zhang X, Ternerot J, Schubert A, Linder S (2020). Induction of ER Stress in Acute Lymphoblastic Leukemia Cells by the Deubiquitinase Inhibitor VLX1570. Int J Mol Sci.

[107] Kurozumi N, Tsujioka T, Ouchida M, Sakakibara K, Nakahara T, Suemori SI, Takeuchi M, Kitanaka A, Shibakura M, Tohyama K (2021). VLX1570 induces apoptosis through the generation of ROS and induction of ER stress on leukemia cell lines. Cancer Sci.

[108] Liu N, Li X, Huang H, Zhao C, Liao S, Yang C, Liu S, Song W, Lu X, Lan X (2014). Clinically used antirheumatic agent auranofin is a proteasomal deubiquitinase inhibitor and inhibits tumor growth. Oncotarget.

[109] Cui XY, Park SH, Park WH (2020). Auranofin inhibits the proliferation of lung cancer cells via necrosis and caspasedependent apoptosis. Oncol Rep.

[110] Li J, Yakushi T, Parlati F, Mackinnon AL, Perez C, Ma Y, Carter KP, Colayco S, Magnuson G, Brown B (2017). Capzimin is a potent and specific inhibitor of proteasome isopeptidase Rpn11. Nat Chem Biol.

[111] Chen J, Dexheimer TS, Ai Y, Liang Q, Villamil MA, Inglese J, Maloney DJ, Jadhav A, Simeonov A, Zhuang Z (2011). Selective and cell-active inhibitors of the USP1/ UAF1 deubiquitinase complex reverse cisplatin resistance in non-small cell lung cancer cells. Chem Biol.

[112] Mistry H, Hsieh G, Buhrlage SJ, Huang M, Park E, Cuny GD, Galinsky I, Stone RM, Gray NS, D'Andrea AD (2013). Small-molecule inhibitors of USP1 target ID1 degradation in leukemic cells. Mol Cancer Ther.

[113] Chuang SJ, Cheng SC, Tang HC, Sun CY, Chou CY (2018). 6-Thioguanine is a noncompetitive and slow binding inhibitor of human deubiquitinating protease USP2. Sci Rep.

[114] Issaeva N, Thomas HD, Djureinovic T, Jaspers JE, Stoimenov I, Kyle S, Pedley N, Gottipati P, Zur R, Sleeth K (2010). 6-thioguanine selectively kills BRCA2-defective tumors and overcomes PARP inhibitor resistance. Can Res.

[115] Litomska A, Ishida K, Dunbar KL, Boettger M, Coyne S, Hertweck C (2018). Enzymatic thioamide formation in a bacterial antimetabolite pathway. Angew Chem Int Ed Engl.

[116] Colland F, Formstecher E, Jacq X, Reverdy C, Planquette C, Conrath S, Trouplin V, Bianchi J, Aushev VN, Camonis J (2009). Small-molecule inhibitor of USP7/HAUSP ubiquitin protease stabilizes and activates p53 in cells. Mol Cancer Ther.

[117] Yang J, Meng C, Weisberg E, Case A, Lamberto I, Magin RS, Adamia S, Wang J, Gray N, Liu S (2020). Inhibition of the deubiquitinase USP10 induces degradation of SYK. Br J Cancer.

[118] Ye M, He J, Zhang J, Liu B, Liu X, Xie L, Wei M, Dong R, Li K, Ma D (2021). USP7 promotes hepatoblastoma progression through activation PI3K/AKT signaling pathway. Cancer Biomark.

[119] He Y, Wang S, Tong J, Jiang S, Yang Y, Zhang Z, Xu Y, Zeng Y, Cao B, Moran MF (2020). The deubiquitinase USP7 stabilizes Maf proteins to promote myeloma cell survival. J Biol Chem.

[120] Peng Y, Liu Y, Gao Y, Yuan B, Qi X, Fu Y, Zhu Q, Cao T, Zhang S, Yin L (2019). USP7 is a novel Deubiquitinase sustaining PLK1 protein stability and regulating chromosome alignment in mitosis. J Exp Clin Cancer Res.

[121] Kategaya L, Di Lello P, Rouge L, Pastor R, Clark KR, Drummond J, Kleinheinz T, Lin E, Upton JP, Prakash S (2017). USP7 small-molecule inhibitors interfere with ubiquitin binding. Nature.

[122] Turnbull AP, Ioannidis S, Krajewski WW, Pinto-Fernandez A, Heride C, Martin ACL, Tonkin LM, Townsend EC, Buker SM, Lancia DR (2017). Molecular basis of USP7 inhibition by selective small-molecule inhibitors. Nature.

[123] Lamberto I, Liu X, Seo HS, Schauer NJ, Iacob RE, Hu W, Das D, Mikhailova T, Weisberg EL, Engen JR (2017). Structure-Guided Development of a Potent and Selective Non-covalent Active-Site Inhibitor of USP7. Cell Chem Biol.

[124] Stolte B, Iniguez AB, Dharia NV, Robichaud AL, Conway AS, Morgan AM, Alexe G, Schauer NJ, Liu X, Bird GH (2018). Genome-scale CRISPR-Cas9 screen identifies druggable dependencies in TP53 wild-type Ewing sarcoma. J Exp Med.

[125] Tian X, Isamiddinova NS, Peroutka RJ, Goldenberg SJ, Mattern MR, Nicholson B, Leach C (2011). Characterization of selective ubiquitin and ubiquitin-like protease inhibitors using a fluorescence-based multiplex assay format. Assay Drug Dev Technol.

[126] Altun M, Kramer HB, Willems LI, McDermott JL, Leach CA, Goldenberg SJ, Kumar KG, Konietzny R, Fischer R, Kogan E (2011). Activity-based chemical proteomics accelerates inhibitor development for deubiquitylating enzymes. Chem Biol.

[127] Shin SB, Kim CH, Jang HR, Yim H (2020). Combination of Inhibitors of USP7 and PLK1 has a Strong Synergism against Paclitaxel Resistance. Int J Mol Sci.

[128] Tavana O, Li D, Dai C, Lopez G, Banerjee D, Kon N, Chen C, Califano A, Yamashiro DJ, Sun H (2016). HAUSP deubiquitinates and stabilizes N-Myc in neuroblastoma. Nat Med.

[129] Ma T, Chen W, Zhi X, Liu H, Zhou Y, Chen BW, Hu L, Shen J, Zheng X, Zhang S (2018). USP9X inhibition improves gemcitabine sensitivity in pancreatic cancer by inhibiting autophagy. Cancer Lett.

[130] Xia X, Huang C, Liao Y, Liu Y, He J, Guo Z, Jiang L, Wang X, Liu J, Huang H (2019). Inhibition of USP14 enhances the sensitivity of breast cancer to enzalutamide. J Exp Clin Cancer Res.

[131] Kurozumi N, Tsujioka T, Ouchida M, Sakakibara K, Nakahara T, Suemori SI, Takeuchi M, Kitanaka A, Shibakura M, Tohyama K (2021). VLX1570 induces apoptosis through the generation of ROS and induction of ER stress on leukemia cell lines. Cancer Sci.

[132] Liu S, Gonzalez-Prieto R, Zhang M, Geurink PP, Kooij R, Iyengar PV, van Dinther M, Bos E, Zhang X, Le Devedec SE (2020). Deubiquitinase Activity Profiling Identifies UCHL1 as a candidate oncoprotein that promotes TGFbeta-induced breast cancer metastasis. Clin Cancer Res.

[133] Schlierf A, Altmann E, Quancard J, Jefferson AB, Assenberg R, Renatus M, Jones M, Hassiepen U, Schaefer M, Kiffe M (2016). Targeted inhibition of the COP9 signalosome for treatment of cancer. Nat Commun.

[134] Song Y, Li S, Ray A, Das DS, Qi J, Samur MK, Tai YT, Munshi N, Carrasco RD, Chauhan D (2017). Blockade of deubiquitylating enzyme Rpn11 triggers apoptosis in multiple myeloma cells and overcomes bortezomib resistance. Oncogene.

[135] Wang L, Li M, Sha B, Hu X, Sun Y, Zhu M, Xu Y, Li P, Wang Y, Guo Y (2021). Inhibition of deubiquitination by PR-619 induces apoptosis and autophagy via ubi-protein aggregation-activated ER stress in oesophageal squamous cell carcinoma. Cell Prolif.

[136] Mirzapoiazova T, Pozhitkov A, Nam A, Mambetsariev I, Nelson MS, Tan YC, Zhang K, Raz D, Singhal S, Nasser MW (2020). Effects of selected deubiquitinating enzyme inhibitors on the proliferation and motility of lung cancer and mesothelioma cell lines. Int J Oncol.

