# Supplementary material for: The emerging role of deubiquitylating enzymes as therapeutic targets in cancer metabolism
Source: Cancer Cell Int. 2022 Mar 20;22:130. doi: 10.1186/s12935-022-02524-y (PMC8935717; doi:10.1186/s12935-022-02524-y)
Supplement: Supplementary file 1 — Additional file 1: Table S1. List of DUBs highly expressed in cancers. [file 12935_2022_2524_MOESM1_ESM.docx]

| **Table S1. List of DUBs highly expressed in cancers.** | | | | |
| --- | --- | --- | --- | --- |
| **DUBs** | **Cacner type** | **Cellular function** | | **references** |
| USP1 | glioblastoma | stemness | | [1] |
|  | osteosarcomas | proliferation, inhibition of differentiation | | [2] |
|  | breast cancer | cell migration | | [3] |
|  | hepatocellular carcinoma | cell cycle | | [4] |
| USP2 | breast cancer | migration and invasion | | [5] |
|  | prostate cancer | proliferation and invasion | | [6] |
|  | gliomas | cancer metabolism | | [7] |
| USP3 | Gastric cancer | metastasis | | [8] |
|  | glioblastomas | migration and invasion | | [9] |
|  | esophageal squamous cell carcinoma | proliferation and metastasis | | [10] |
| USP4 | lung cancer | stemness | | [11] |
|  | melanoma | epithelial-mesenchymal transition | | [12] |
|  | colorectal cancer | cell growth and metastasis | | [13] |
| USP5 | hepatocellular carcinoma | epithelial-mesenchymal transition | | [14] |
|  | non-small cell lung cancer | cell proliferation | | [15] |
|  | glioblastoma multiforme | proliferation maintenance | | [16] |
| USP6 | ewing sarcoma | tumor suppressive | | [17] |
|  | colon cancer | invasion and metastasis | | [18] |
|  | osteosarcoma | cell viability and migratation | | [19] |
| USP7 | oral squamous cell carcinoma | proliferation and invasion | | [20] |
|  | osteosarcoma | epithelial‑mesenchymal transition | | [21] |
|  | hepatocellular carcinoma | proliferation, migration, and invasion | | [22] |
| USP8 | cervical squamous cell carcinoma | proliferation, migration, and invasion | | [23] |
|  | gastric cancer | proliferation and migration | | [24] |
| USP9X | pancreatic ductal adenocarcinoma | cell migration and invasion | | [25] |
|  | non-small cell lung cancer | progression and metastasis | | [26] |
|  | hepatocellular carcinoma | cell proliferation | | [27] |
| USP10 | lung cancer | | xenografts growth | [28] |
|  | hepatocellular carcinoma | | proliferation | [29] |
|  | colorectal cancer | | tumor progression and metastasis | [30] |
| USP11 | hepatocellular carcinoma | | proliferation and metastasis | [31] |
|  | gastric cancer | | growth and migration | [32] |
|  | colorectal cancer | | proliferation and metastasis | [33] |
|  | ovarian cancer | | epithelial‑to‑mesenchymal transition | [34] |
| USP12 | multiple myeloma | | pro-survival autophagy and drug resistance | [35] |
|  | breast cancer | | promotion of angiogenesis | [36] |
|  | hepatocellular carcinoma | | proliferation, cell cycle arrest | [37] |
| USP13 | gastric cancer | | epithelial-mesenchymal transition and metastasis | [38] |
|  | hepatocellular carcinoma | | tumor growth and metastasis | [39] |
|  | lung cancer | | promotion of proliferation | [40] |
|  | cervical cancer | | promotion of proliferation | [41] |
| USP14 | hepatocellular carcinoma | | cell proliferation, invasion, migration, and vascular mimicry formation | [42] |
|  | epithelial ovarian cancer | | cellular proliferation and apoptosis | [43] |
|  | non-small cell lung cancer | | migration | [44] |
|  | pancreatic ductal adenocarcinoma | | proliferation and metastasis | [45] |
| USP15 | gastric cancer | | proliferation, invasion and epithelial-mesenchymal transition | [46] |
|  | hepatocellular carcinoma | | proliferation and apoptosis | [47] |
|  | multiple myeloma | | apoptosis | [48] |
|  | acute myeloid leukemia | | cellular redox | [49] |
| USP16 | prostate cancer | | proliferation | [50] |
| USP17 | non-small cell lung cancer | | migration and invasion | [51-53] |
|  | osteosarcoma | | cell proliferation, metastasis, and epithelial-mesenchymal transition | [54] |
|  | breast cancer | | epithelial-mesenchymal transition | [55] |
| USP18 | esophageal squamous cell carcinomas | | metastasis | [56] |
|  | colorectal carcinoma | | proliferation, migration and invasion | [57] |
|  | glioblastoma | | epithelial-mesenchymal transition | [58] |
|  | pancreatic cancer | | cell cycle progression | [59] |
| USP19 | gastric cancer | | proliferation, anti-apoptotic and metastasis | [60] |
| USP20 | breast cancer | | migration, invasion and metastasis | [61, 62] |
|  | lung cancer | | migration and response to chemotherapy | [61] |
|  | colon cancer | | proliferation |  |
|  | ovarian cancer | | proliferation, response to chemotherapy |  |
| USP21 | colorectal cancer | | metastasis | [63] |
|  | pancreatic ductal adenocarcinoma | | stemness | [64] |
|  | cholangiocarcinoma | | proliferation and migration | [65] |
|  | gastric cancer | | proliferation, migration, invasion and stemness | [66] |
|  | non-small-cell lung cancer | | proliferation, migration and invasion | [67] |
| USP22 | breast cancer | | cell growth and proliferation | [68] |
|  | hepatocellular carcinoma | | stemness | [69] |
|  | gastric cancer | | Proliferation and metastasis | [70] |
|  | glioma | | proliferation, migration and invasion | [71] |
| USP26 | esophageal squamous cell carcinoma | | migration and invasion | [72] |
| USP27X | osteosarcoma | | cell viability and migratation | [19] |
| USP28 | pancreatic cancer | | cell cycle progression | [73] |
|  | non-small-cell lung cancer | | cell growth | [74] |
|  | colorectal cancer | | cell viability and aerobic glycolysis | [75] |
|  | gastric cancer | | proliferation and metastasis | [76] |
| USP29 | colorectal carcinoma | | cell proliferation and cell-cycle progression | [77] |
| USP32 | gastric carcinoma | | cell growth, metastasis and chemoresistance | [78] |
|  | small cell lung cancer | | migration and cell cycle progression | [79] |
|  | epithelial ovarian cancer | | epithelial mesenchymal transition | [80] |
| USP33 | retinoblastoma | | proliferation and reduction of apoptosis | [81] |
|  | hepatocellular carcinoma | | invasion and metastasis | [82] |
|  | prostate cancer | | Proliferation and cell cycle | [83] |
| USP34 | laryngeal squamous cell carcinoma | | cell survival and drug resistance | [84] |
|  | pancreatic cancer | | proliferation, migration and apoptosis | [85] |
| USP35 | non-small cell lung cancer | | mitigation of apoptosis | [86] |
|  | ovarian cancer | | interferon signaling | [87] |
|  | estrogen receptor α positive breast cancer | | tumorigenesis | [88] |
| USP37 | lung cancer | | proliferation | [89] |
|  | breast cancer | | stemness, cell invasion and epithelial-mesenchymal transition, drug sensitivity | [90] |
| USP39 | osteosarcoma | | proliferation, cell cycle regulation, apoptosis and tumorigenesis | [91] |
|  | renal cell carcinoma | | malignant proliferation and angiogenesis | [92] |
|  | esophageal squamous cell carcinoma | | tumorigenesis | [93] |
| USP41 | osteosarcoma | | migration | [19] |
|  | lung cancer | | proliferation and migration | [94] |
|  | breast cancer | | growth, proliferation and invasion | [95] |
| USP42 | gastric cancer | | proliferation, cell cycle and invasive capacity | [96] |
| USP43 | osteosarcoma | | cell viability | [19] |
|  | colorectal cancer | | proliferation and metastasis | [97] |
|  | breast cancer | | cell cycle and epithelial mesenchymal transition | [98] |
| USP44 | gastric cancer | | DNA aneuploidy | [99] |
| USP46 | esophageal squamous cell carcinoma | | metastasis | [100] |
| USP47 | colorectal cancer | | proliferation, epithelial-mesenchymal transition and metastasis | [101, 102] |
|  | breast cancer | | epithelial mesenchymal transition | [103] |
|  | gastric cancer | | chemoresistance and Cell viability | [104] |
| USP54 | colorectal cancer | | tumorigenesis | [105] |

| ATXN3 | anaplastic thyroid carcinoma | proliferation and metastasis | [106] |
| --- | --- | --- | --- |
|  | breast cancer | stemness and metastasis | [107, 108] |
|  | colon cancer | cell viability and migration | [109] |
| BRCC3 | bladder cancer | proliferation, viability and migration | [110] |
|  | Gastric cancer | proliferation, migration and angiogenesis. | [111] |
|  | cervical cancer | cell viability, invasion and migration | [112] |
| CSN5 | hepatocellular carcinoma | glycolysis | [113] |
|  | breast Cancer | proliferation and chemoresistance | [114-116] |
|  | renal cell carcinoma | migration and invasion | [117] |
|  | serous ovarian cancer | cell cycle | [118] |
| JOSD1 | head and neck squamous cell carcinoma | proliferation and chemoresistance | [119] |
|  | gynaecological cancer | chemoresistance | [120] |
| JOSD2 | small cell lung cancer | cancer metabolism | [121] |
| OTUB1 | bladder cancer | proliferation, viability, and migration | [122] |
|  | glioma | stemness | [123] |
|  | prostate cancer | proliferation and metastasis | [124] |
|  | colorectal cancer | metastasis | [125] |
| OTUB2 | colorectal cancer | glycolysis | [126] |
|  | Gastric Cancer | stemness | [127] |
|  | Endometrial Cancer | homologous recombination repair | [128] |
|  | non-small cell lung cancer | Warburg effect | [129] |
| OTUD7B | Breast Cancer | proliferation and metastasis | [130, 131] |
|  | Lung Cancer | proliferation and metastasis | [132] |
| PSMD14 | ovarian cancer | glycolysis | [133] |
|  | esophageal squamous cell carcinoma | motility and stemness | [134, 135] |
|  | head and neck squamous cell carcinoma | growth, chemoresistance and stemness | [136] |
|  | colorectal cancer | cancer stemness/chemoresistance | [137] |
| STAMBP | lung adenocarcinoma | metastasis | [138] |
|  | Head and Neck Squamous Cell Carcinoma | proliferation, migration, and invasion | [139] |
| STAMBPL1 | gastric cancer | proliferation | [140] |
|  | prostate cancer | survival | [141] |
| UCHL1 | High-grade serous ovarian cancer | proliferation | [142] |
|  | Uterine Serous Cancer | proliferation and cell Cycle | [143] |
| UCHL3 | pancreatic cancer | aerobic glycolysis | [144] |
|  | non-small cell lung cancers | stemness | [145] |
|  | ovarian cancer | proliferation, viability and migration | [146] |
| UCHL5 | lung adenocarcinoma | Proliferation and cell cycle | [147] |
|  | Endometrial Cancer | proliferation | [148] |
|  | multiple myeloma | survival | [149] |
| UCK2 | melanoma | metastasis | [150] |
|  | lung cancer | proliferation and migration | [151] |
|  | hepatocellular carcinoma | metastasis and proliferation | [152-154] |
| YOD1 | liver cancer | hippo signaling | [155] |
| ZRANB1 | colorectal cancer | stemness | [156] |
|  | breast Cancer | proliferation | [157] |

1. Lee JK, Chang N, Yoon Y, Yang H, Cho H, Kim E, Shin Y, Kang W, Oh YT, Mun GI *et al*: **USP1 targeting impedes GBM growth by inhibiting stem cell maintenance and radioresistance**. *Neuro Oncol* 2016, **18**(1):37-47.

2. Williams SA, Maecker HL, French DM, Liu J, Gregg A, Silverstein LB, Cao TC, Carano RA, Dixit VM: **USP1 deubiquitinates ID proteins to preserve a mesenchymal stem cell program in osteosarcoma**. *Cell* 2011, **146**(6):918-930.

3. Ma A, Tang M, Zhang L, Wang B, Yang Z, Liu Y, Xu G, Wu L, Jing T, Xu X *et al*: **USP1 inhibition destabilizes KPNA2 and suppresses breast cancer metastasis**. *Oncogene* 2019, **38**(13):2405-2419.

4. Zhao Y, Xue C, Xie Z, Ouyang X, Li L: **Comprehensive analysis of ubiquitin-specific protease 1 reveals its importance in hepatocellular carcinoma**. *Cell proliferation* 2020, **53**(10):e12908.

5. Qu Q, Mao Y, Xiao G, Fei X, Wang J, Zhang Y, Liu J, Cheng G, Chen X, Wang J *et al*: **USP2 promotes cell migration and invasion in triple negative breast cancer cell lines**. *Tumour biology : the journal of the International Society for Oncodevelopmental Biology and Medicine* 2015, **36**(7):5415-5423.

6. Benassi B, Flavin R, Marchionni L, Zanata S, Pan Y, Chowdhury D, Marani M, Strano S, Muti P, Blandino G *et al*: **MYC is activated by USP2a-mediated modulation of microRNAs in prostate cancer**. *Cancer discovery* 2012, **2**(3):236-247.

7. Tao BB, He H, Shi XH, Wang CL, Li WQ, Li B, Dong Y, Hu GH, Hou LJ, Luo C *et al*: **Up-regulation of USP2a and FASN in gliomas correlates strongly with glioma grade**. *J Clin Neurosci* 2013, **20**(5):717-720.

8. Wu X, Wang H, Zhu D, Chai Y, Wang J, Dai W, Xiao Y, Tang W, Li J, Hong L *et al*: **USP3 promotes gastric cancer progression and metastasis by deubiquitination-dependent COL9A3/COL6A5 stabilisation**. *Cell death & disease* 2021, **13**(1):10.

9. Fan L, Chen Z, Wu X, Cai X, Feng S, Lu J, Wang H, Liu N: **Ubiquitin-Specific Protease 3 Promotes Glioblastoma Cell Invasion and Epithelial-Mesenchymal Transition via Stabilizing Snail**. *Molecular cancer research : MCR* 2019, **17**(10):1975-1984.

10. Shi K, Zhang JZ, Yang L, Li NN, Yue Y, Du XH, Zhang XZ, Lu YC, Guo D: **Protein deubiquitylase USP3 stabilizes Aurora A to promote proliferation and metastasis of esophageal squamous cell carcinoma**. *BMC Cancer* 2021, **21**(1):1196.

11. Li F, Hu Q, He T, Xu J, Yi Y, Xie S, Ding L, Fu M, Guo R, Xiao ZJ *et al*: **The Deubiquitinase USP4 Stabilizes Twist1 Protein to Promote Lung Cancer Cell Stemness**. *Cancers (Basel)* 2020, **12**(6).

12. Guo W, Ma J, Pei T, Zhao T, Guo S, Yi X, Liu Y, Wang S, Zhu G, Jian Z *et al*: **Up-regulated deubiquitinase USP4 plays an oncogenic role in melanoma**. *J Cell Mol Med* 2018, **22**(5):2944-2954.

13. Xing C, Lu XX, Guo PD, Shen T, Zhang S, He XS, Gan WJ, Li XM, Wang JR, Zhao YY *et al*: **Ubiquitin-Specific Protease 4-Mediated Deubiquitination and Stabilization of PRL-3 Is Required for Potentiating Colorectal Oncogenesis**. *Cancer research* 2016, **76**(1):83-95.

14. Meng J, Ai X, Lei Y, Zhong W, Qian B, Qiao K, Wang X, Zhou B, Wang H, Huai L *et al*: **USP5 promotes epithelial-mesenchymal transition by stabilizing SLUG in hepatocellular carcinoma**. *Theranostics* 2019, **9**(2):573-587.

15. Ma X, Qi W, Pan H, Yang F, Deng J: **Overexpression of USP5 contributes to tumorigenesis in non-small cell lung cancer via the stabilization of β-catenin protein**. *Am J Cancer Res* 2018, **8**(11):2284-2295.

16. Li G, Yang T, Chen Y, Bao J, Wu D, Hu X, Feng C, Xu L, Li M, Li G *et al*: **USP5 Sustains the Proliferation of Glioblastoma Through Stabilization of CyclinD1**. *Front Pharmacol* 2021, **12**:720307.

17. Henrich IC, Jain K, Young R, Quick L, Lindsay JM, Park DH, Oliveira AM, Blobel GA, Chou MM: **Ubiquitin-Specific Protease 6 Functions as a Tumor Suppressor in Ewing Sarcoma through Immune Activation**. *Cancer Res* 2021, **81**(8):2171-2183.

18. Zeng H, Yuan F, Mi Y, Xian G, Qin C, Zhang D: **As an independent prognostic factor, USP6 promotes the invasion and metastasis of colon cancer**. *Biochem Biophys Res Commun* 2018, **505**(3):816-822.

19. Lavaud M, Mullard M, Tesfaye R, Amiaud J, Legrand M, Danieau G, Brion R, Morice S, Regnier L, Dupuy M *et al*: **Overexpression of the Ubiquitin Specific Proteases USP43, USP41, USP27x and USP6 in Osteosarcoma Cell Lines: Inhibition of Osteosarcoma Tumor Growth and Lung Metastasis Development by the USP Antagonist PR619**. *Cells* 2021, **10**(9).

20. Yang X, Jin J, Yang J, Zhou L, Mi S, Qi G: **Expression of Ubiquitin-specific protease 7 in oral squamous cell carcinoma promotes tumor cell proliferation and invasion**. *Genetics and molecular biology* 2021, **44**(4):e20210058.

21. Zeng Q, Li Z, Zhao X, Guo L, Yu C, Qin J, Zhang S, Zhang Y, Yang X: **Ubiquitin‑specific protease 7 promotes osteosarcoma cell metastasis by inducing epithelial‑mesenchymal transition**. *Oncol Rep* 2019, **41**(1):543-551.

22. Wang X, Zhang Q, Wang Y, Zhuang H, Chen B: **Clinical Significance of Ubiquitin Specific Protease 7 (USP7) in Predicting Prognosis of Hepatocellular Carcinoma and its Functional Mechanisms**. *Medical science monitor : international medical journal of experimental and clinical research* 2018, **24**:1742-1750.

23. Yan M, Zhao C, Wei N, Wu X, Cui J, Xing Y: **High Expression of Ubiquitin-Specific Protease 8 (USP8) Is Associated with Poor Prognosis in Patients with Cervical Squamous Cell Carcinoma**. *Medical science monitor : international medical journal of experimental and clinical research* 2018, **24**:4934-4943.

24. Sun J, Shen D, Zheng Y, Ren H, Liu H, Chen X, Gao Y: **USP8 Inhibitor Suppresses HER-2 Positive Gastric Cancer Cell Proliferation and Metastasis via the PI3K/AKT Signaling Pathway**. *Onco Targets Ther* 2020, **13**:9941-9952.

25. Liu L, Yao D, Zhang P, Ding W, Zhang X, Zhang C, Gong S, Zhang Y, Wang J, Sun T *et al*: **Deubiquitinase USP9X promotes cell migration, invasion and inhibits apoptosis of human pancreatic cancer**. *Oncol Rep* 2017, **38**(6):3531-3537.

26. Wang Y, Liu Y, Yang B, Cao H, Yang CX, Ouyang W, Zhang SM, Yang GF, Zhou FX, Zhou YF *et al*: **Elevated expression of USP9X correlates with poor prognosis in human non-small cell lung cancer**. *Journal of thoracic disease* 2015, **7**(4):672-679.

27. Chen MY, Li ZP, Sun ZN, Ma M: **USP9X promotes the progression of hepatocellular carcinoma by regulating beta-catenin**. *Irish journal of medical science* 2020, **189**(3):865-871.

28. Hu C, Zhang M, Moses N, Hu CL, Polin L, Chen W, Jang H, Heyza J, Malysa A, Caruso JA *et al*: **The USP10-HDAC6 axis confers cisplatin resistance in non-small cell lung cancer lacking wild-type p53**. *Cell Death Dis* 2020, **11**(5):328.

29. Zhu H, Yan F, Yuan T, Qian M, Zhou T, Dai X, Cao J, Ying M, Dong X, He Q *et al*: **USP10 Promotes Proliferation of Hepatocellular Carcinoma by Deubiquitinating and Stabilizing YAP/TAZ**. *Cancer Res* 2020, **80**(11):2204-2216.

30. Li B, Qi ZP, He DL, Chen ZH, Liu JY, Wong MW, Zhang JW, Xu EP, Shi Q, Cai SL *et al*: **NLRP7 deubiquitination by USP10 promotes tumor progression and tumor-associated macrophage polarization in colorectal cancer**. *J Exp Clin Cancer Res* 2021, **40**(1):126.

31. Zhang C, Xie C, Wang X, Huang Y, Gao S, Lu J, Lu Y, Zhang S: **Aberrant USP11 expression regulates NF90 to promote proliferation and metastasis in hepatocellular carcinoma**. *Am J Cancer Res* 2020, **10**(5):1416-1428.

32. Liu H, Liu M, He B, Li Q: **Inhibition of USP11 sensitizes gastric cancer to chemotherapy via suppressing RhoA and Ras-mediated signaling pathways**. *Clinics and research in hepatology and gastroenterology* 2021, **46**(1):101779.

33. Huang YY, Zhang CM, Dai YB, Lin JG, Lin N, Huang ZX, Xu TW: **USP11 facilitates colorectal cancer proliferation and metastasis by regulating IGF2BP3 stability**. *American journal of translational research* 2021, **13**(2):480-496.

34. Wang W, Wang J, Yan H, Zhang K, Liu Y: **Upregulation of USP11 promotes epithelial‑to‑mesenchymal transition by deubiquitinating Snail in ovarian cancer**. *Oncol Rep* 2019, **41**(3):1739-1748.

35. Li H, Roy M, Liang L, Cao W, Hu B, Li Y, Xiao X, Wang H, Ye M, Sun S *et al*: **Deubiquitylase USP12 induces pro-survival autophagy and bortezomib resistance in multiple myeloma by stabilizing HMGB1**. *Oncogene* 2022.

36. Sheng B, Wei Z, Wu X, Li Y, Liu Z: **USP12 promotes breast cancer angiogenesis by maintaining midkine stability**. *Cell Death Dis* 2021, **12**(11):1074.

37. Liu C, Li X, Feng G, Cao M, Liu F, Zhang G, Lu Y: **Downregulation of USP12 inhibits tumor growth via the p38/MAPK pathway in hepatocellular carcinoma**. *Mol Med Rep* 2020, **22**(6):4899-4908.

38. Zhang T, Zheng J, Qiao L, Zhao W: **Deubiquitinase USP13 promotes the epithelial-mesenchymal transition and metastasis in gastric cancer by maintaining Snail protein**. *Pathology, research and practice* 2021, **229**:153705.

39. Gao S, Chen T, Li L, Liu X, Liu Y, Zhao J, Lu Q, Zeng Z, Xu Q, Huang D *et al*: **Hypoxia-Inducible Ubiquitin Specific Peptidase 13 Contributes to Tumor Growth and Metastasis via Enhancing the Toll-Like Receptor 4/Myeloid Differentiation Primary Response Gene 88/Nuclear Factor-κB Pathway in Hepatocellular Carcinoma**. *Front Cell Dev Biol* 2020, **8**:587389.

40. Wu Y, Zhang Y, Liu C, Zhang Y, Wang D, Wang S, Wu Y, Liu F, Li Q, Liu X *et al*: **Amplification of USP13 drives non-small cell lung cancer progression mediated by AKT/MAPK signaling**. *Biomedicine & pharmacotherapy = Biomedecine & pharmacotherapie* 2019, **114**:108831.

41. Morgan EL, Patterson MR, Barba-Moreno D, Scarth JA, Wilson A, Macdonald A: **The deubiquitinase (DUB) USP13 promotes Mcl-1 stabilisation in cervical cancer**. *Oncogene* 2021, **40**(11):2112-2129.

42. Lv C, Wang S, Lin L, Wang C, Zeng K, Meng Y, Sun G, Wei S, Liu Y, Zhao Y: **USP14 maintains HIF1-α stabilization via its deubiquitination activity in hepatocellular carcinoma**. *Cell Death Dis* 2021, **12**(9):803.

43. Wang Y, Wang J, Zhong J, Deng Y, Xi Q, He S, Yang S, Jiang L, Huang M, Tang C *et al*: **Ubiquitin-specific protease 14 (USP14) regulates cellular proliferation and apoptosis in epithelial ovarian cancer**. *Medical oncology (Northwood, London, England)* 2015, **32**(1):379.

44. Yan G, Liu NA, Wang J, Tian J, Liu H, Li S, Liu W, Li X, Li K, Wang H: **Deubiquitylation and stabilization of Acf7 by ubiquitin carboxylterminal hydrolase 14 (USP14) is critical for NSCLC migration**. *Journal of biosciences* 2021, **46**.

45. Hang C, Gong C, Fang Y, Chen L, Zhu J: **Ubiquitin-specific protease 14 (USP14) promotes proliferation and metastasis in pancreatic ductal adenocarcinoma**. *Journal of molecular histology* 2021, **52**(2):187-196.

46. Zhong M, Zhou L, Fang Z, Yao YY, Zou JP, Xiong JP, Xiang XJ, Deng J: **Ubiquitin-specific protease 15 contributes to gastric cancer progression by regulating the Wnt/β-catenin signaling pathway**. *World journal of gastroenterology* 2021, **27**(26):4221-4235.

47. Yao XQ, Li L, Piao LZ, Zhang GJ, Huang XZ, Wang Y, Liang ZL: **Overexpression of Ubiquitin-Specific Protease15 (USP15) Promotes Tumor Growth and Inhibits Apoptosis and Correlated With Poor Disease-Free Survival in Hepatocellular Carcinoma**. *Technology in cancer research & treatment* 2020, **19**:1533033820967455.

48. Zhou L, Jiang H, Du J, Li L, Li R, Lu J, Fu W, Hou J: **USP15 inhibits multiple myeloma cell apoptosis through activating a feedback loop with the transcription factor NF-κBp65**. *Exp Mol Med* 2018, **50**(11):1-12.

49. Niederkorn M, Ishikawa C, K MH, Bartram J, Stepanchick E, J RB, A EC-C, Bolanos LC, Uible E, Choi K *et al*: **The deubiquitinase USP15 modulates cellular redox and is a therapeutic target in acute myeloid leukemia**. *Leukemia* 2021.

50. Ge J, Yu W, Li J, Ma H, Wang P, Zhou Y, Wang Y, Zhang J, Shi G: **USP16 regulates castration-resistant prostate cancer cell proliferation by deubiquitinating and stablizing c-Myc**. *J Exp Clin Cancer Res* 2021, **40**(1):59.

51. McFarlane C, McFarlane S, Paul I, Arthur K, Scheaff M, Kerr K, Stevenson M, Fennell DA, Johnston JA: **The deubiquitinating enzyme USP17 is associated with non-small cell lung cancer (NSCLC) recurrence and metastasis**. *Oncotarget* 2013, **4**(10):1836-1843.

52. Zhang S, Yuan J, Zheng R: **Suppression of Ubiquitin-Specific Peptidase 17 (USP17) Inhibits Tumorigenesis and Invasion in Non-Small Cell Lung Cancer Cells**. *Oncology research* 2016, **24**(4):263-269.

53. Zhang S, Xu Z, Yuan J, Chen H: **Ubiquitin-specific peptidase 17 promotes cisplatin resistance via PI3K/AKT activation in non-small cell lung cancer**. *Oncol Lett* 2020, **20**(1):67-74.

54. Song C, Liu W, Li J: **USP17 is upregulated in osteosarcoma and promotes cell proliferation, metastasis, and epithelial-mesenchymal transition through stabilizing SMAD4**. *Tumour Biol* 2017, **39**(7):1010428317717138.

55. Wu Y, Wang Y, Lin Y, Liu Y, Wang Y, Jia J, Singh P, Chi YI, Wang C, Dong C *et al*: **Dub3 inhibition suppresses breast cancer invasion and metastasis by promoting Snail1 degradation**. *Nat Commun* 2017, **8**:14228.

56. Song C, Peng J, Wei Y, Shao J, Chen X, Zhang X, Xu J: **USP18 promotes tumor metastasis in esophageal squamous cell carcinomas via deubiquitinating ZEB1**. *Exp Cell Res* 2021, **409**(1):112884.

57. Huang F, Zheng C, Huang L, Lin C, Wang J: **USP18 directly regulates Snail1 protein through ubiquitination pathway in colorectal cancer**. *Cancer Cell Int* 2020, **20**:346.

58. Cai X, Feng S, Zhang J, Qiu W, Qian M, Wang Y: **USP18 deubiquitinates and stabilizes Twist1 to promote epithelial-mesenchymal transition in glioblastoma cells**. *Am J Cancer Res* 2020, **10**(4):1156-1169.

59. Feng L, Wang K, Tang P, Chen S, Liu T, Lei J, Yuan R, Hu Z, Li W, Yu X: **Deubiquitinase USP18 promotes the progression of pancreatic cancer via enhancing the Notch1-c-Myc axis**. *Aging (Albany NY)* 2020, **12**(19):19273-19292.

60. Dong Z, Guo S, Wang Y, Zhang J, Luo H, Zheng G, Yang D, Zhang T, Yan L, Song L *et al*: **USP19 Enhances MMP2/MMP9-Mediated Tumorigenesis in Gastric Cancer**. *Onco Targets Ther* 2020, **13**:8495-8510.

61. Wu C, Luo K, Zhao F, Yin P, Song Y, Deng M, Huang J, Chen Y, Li L, Lee S *et al*: **USP20 positively regulates tumorigenesis and chemoresistance through β-catenin stabilization**. *Cell Death Differ* 2018, **25**(10):1855-1869.

62. Li W, Shen M, Jiang YZ, Zhang R, Zheng H, Wei Y, Shao ZM, Kang Y: **Deubiquitinase USP20 promotes breast cancer metastasis by stabilizing SNAI2**. *Genes & development* 2020, **34**(19-20):1310-1315.

63. Yun SI, Hong HK, Yeo SY, Kim SH, Cho YB, Kim KK: **Ubiquitin-Specific Protease 21 Promotes Colorectal Cancer Metastasis by Acting as a Fra-1 Deubiquitinase**. *Cancers (Basel)* 2020, **12**(1).

64. Hou P, Ma X, Zhang Q, Wu CJ, Liao W, Li J, Wang H, Zhao J, Zhou X, Guan C *et al*: **USP21 deubiquitinase promotes pancreas cancer cell stemness via Wnt pathway activation**. *Genes & development* 2019, **33**(19-20):1361-1366.

65. Zhou P, Song T, Sun C, He N, Cheng Q, Xiao X, Ran J, Liu M, Xie S: **USP21 upregulation in cholangiocarcinoma promotes cell proliferation and migration in a deubiquitinase-dependent manner**. *Asia-Pacific journal of clinical oncology* 2021, **17**(6):471-477.

66. Guo Q, Shi D, Lin L, Li H, Wei Y, Li B, Wu D: **De-Ubiquitinating Enzymes USP21 Regulate MAPK1 Expression by Binding to Transcription Factor GATA3 to Regulate Tumor Growth and Cell Stemness of Gastric Cancer**. *Front Cell Dev Biol* 2021, **9**:641981.

67. Xu P, Xiao H, Yang Q, Hu R, Jiang L, Bi R, Jiang X, Wang L, Mei J, Ding F *et al*: **The USP21/YY1/SNHG16 axis contributes to tumor proliferation, migration, and invasion of non-small-cell lung cancer**. *Exp Mol Med* 2020, **52**(1):41-55.

68. Wang S, Zhong X, Wang C, Luo H, Lin L, Sun H, Sun G, Zeng K, Zou R, Liu W *et al*: **USP22 positively modulates ERα action via its deubiquitinase activity in breast cancer**. *Cell Death Differ* 2020, **27**(11):3131-3145.

69. Ling S, Shan Q, Zhan Q, Ye Q, Liu P, Xu S, He X, Ma J, Xiang J, Jiang G *et al*: **USP22 promotes hypoxia-induced hepatocellular carcinoma stemness by a HIF1α/USP22 positive feedback loop upon TP53 inactivation**. *Gut* 2020, **69**(7):1322-1334.

70. Liu H, Liu N, Zhao Y, Zhu X, Wang C, Liu Q, Gao C, Zhao X, Li J: **Oncogenic USP22 supports gastric cancer growth and metastasis by activating c-Myc/NAMPT/SIRT1-dependent FOXO1 and YAP signaling**. *Aging (Albany NY)* 2019, **11**(21):9643-9660.

71. Liang J, Zhang XL, Li S, Xie S, Wang WF, Yu RT: **Ubiquitin-specific protease 22 promotes the proliferation, migration and invasion of glioma cells**. *Cancer Biomark* 2018, **23**(3):381-389.

72. Li L, Zhou H, Zhu R, Liu Z: **USP26 promotes esophageal squamous cell carcinoma metastasis through stabilizing Snail**. *Cancer Lett* 2019, **448**:52-60.

73. Chen L, Xu Z, Li Q, Feng Q, Zheng C, Du Y, Yuan R, Peng X: **USP28 facilitates pancreatic cancer progression through activation of Wnt/β-catenin pathway via stabilising FOXM1**. *Cell Death Dis* 2021, **12**(10):887.

74. Li P, Huang Z, Wang J, Chen W, Huang J: **Ubiquitin-specific peptidase 28 enhances STAT3 signaling and promotes cell growth in non-small-cell lung cancer**. *Onco Targets Ther* 2019, **12**:1603-1611.

75. Liu Z, Chen M, Xu X, Zhang L, Pan Y, Chen D: **USP28 promotes aerobic glycolysis of colorectal cancer by increasing stability of FOXC1**. *Acta Biochim Pol* 2021, **68**(4):633-639.

76. Zhao LJ, Zhang T, Feng XJ, Chang J, Suo FZ, Ma JL, Liu YJ, Liu Y, Zheng YC, Liu HM: **USP28 contributes to the proliferation and metastasis of gastric cancer**. *J Cell Biochem* 2018.

77. Chandrasekaran AP, Suresh B, Sarodaya N, Ko NR, Oh SJ, Kim KS, Ramakrishna S: **Ubiquitin Specific Protease 29 Functions as an Oncogene Promoting Tumorigenesis in Colorectal Carcinoma**. *Cancers (Basel)* 2021, **13**(11).

78. Dou N, Hu Q, Li L, Wu Q, Li Y, Gao Y: **USP32 promotes tumorigenesis and chemoresistance in gastric carcinoma via upregulation of SMAD2**. *Int J Biol Sci* 2020, **16**(9):1648-1657.

79. Hu W, Wei H, Li K, Li P, Lin J, Feng R: **Downregulation of USP32 inhibits cell proliferation, migration and invasion in human small cell lung cancer**. *Cell proliferation* 2017, **50**(4).

80. Nakae A, Kodama M, Okamoto T, Tokunaga M, Shimura H, Hashimoto K, Sawada K, Kodama T, Copeland NG, Jenkins NA *et al*: **Ubiquitin specific peptidase 32 acts as an oncogene in epithelial ovarian cancer by deubiquitylating farnesyl-diphosphate farnesyltransferase 1**. *Biochem Biophys Res Commun* 2021, **552**:120-127.

81. Wang H, Liu Z, Sun Z, Zhou D, Mao H, Deng G: **Ubiquitin specific peptidase 33 promotes cell proliferation and reduces apoptosis through regulation of the SP1/PI3K/AKT pathway in retinoblastoma**. *Cell Cycle* 2021, **20**(19):2066-2076.

82. Gan Q, Shao J, Cao Y, Lei J, Xie P, Ge J, Hu G: **USP33 regulates c-Met expression by deubiquitinating SP1 to facilitate metastasis in hepatocellular carcinoma**. *Life Sci* 2020, **261**:118316.

83. Ding T, Zhu Y, Jin H, Zhang P, Guo J, Zheng J: **Circular RNA circ_0057558 Controls Prostate Cancer Cell Proliferation Through Regulating miR-206/USP33/c-Myc Axis**. *Front Cell Dev Biol* 2021, **9**:644397.

84. Dai WL, Yuan SX, Cao JP: **The deubiquitinase USP34 stabilizes SOX2 and induces cell survival and drug resistance in laryngeal squamous cell carcinoma**. *The Kaohsiung journal of medical sciences* 2020, **36**(12):983-989.

85. Lin C, Xia J, Gu Z, Meng Y, Gao D, Wei S: **Downregulation of USP34 Inhibits the Growth and Migration of Pancreatic Cancer Cells via Inhibiting the PRR11**. *Onco Targets Ther* 2020, **13**:1471-1480.

86. Wang W, Wang M, Xiao Y, Wang Y, Ma L, Guo L, Wu X, Lin X, Zhang P: **USP35 mitigates endoplasmic reticulum stress-induced apoptosis by stabilizing RRBP1 in non-small cell lung cancer**. *Molecular oncology* 2021.

87. Zhang J, Chen Y, Chen X, Zhang W, Zhao L, Weng L, Tian H, Wu Z, Tan X, Ge X *et al*: **Deubiquitinase USP35 restrains STING-mediated interferon signaling in ovarian cancer**. *Cell Death Differ* 2021, **28**(1):139-155.

88. Cao J, Wu D, Wu G, Wang Y, Ren T, Wang Y, Lv Y, Sun W, Wang J, Qian C *et al*: **USP35, regulated by estrogen and AKT, promotes breast tumorigenesis by stabilizing and enhancing transcriptional activity of estrogen receptor α**. *Cell Death Dis* 2021, **12**(6):619.

89. Pan J, Deng Q, Jiang C, Wang X, Niu T, Li H, Chen T, Jin J, Pan W, Cai X *et al*: **USP37 directly deubiquitinates and stabilizes c-Myc in lung cancer**. *Oncogene* 2015, **34**(30):3957-3967.

90. Qin T, Li B, Feng X, Fan S, Liu L, Liu D, Mao J, Lu Y, Yang J, Yu X *et al*: **Abnormally elevated USP37 expression in breast cancer stem cells regulates stemness, epithelial-mesenchymal transition and cisplatin sensitivity**. *J Exp Clin Cancer Res* 2018, **37**(1):287.

91. Gan Z, Han K, Lin S, Hu H, Shen Z, Min D: **Knockdown of ubiquitin-specific peptidase 39 inhibited the growth of osteosarcoma cells and induced apoptosis in vitro**. *Biological research* 2017, **50**(1):15.

92. Pan XW, Xu D, Chen WJ, Chen JX, Chen WJ, Ye JQ, Gan SS, Zhou W, Song X, Shi L *et al*: **USP39 promotes malignant proliferation and angiogenesis of renal cell carcinoma by inhibiting VEGF-A(165b) alternative splicing via regulating SRSF1 and SRPK1**. *Cancer Cell Int* 2021, **21**(1):486.

93. Zhao Y, Geng H, Liu G, Ji Q, Cheng X, Li X, Liu W, Thorne RF, Zhang R, Liu X: **The Deubiquitinase USP39 Promotes ESCC Tumorigenesis Through Pre-mRNA Splicing of the mTORC2 Component Rictor**. *Front Oncol* 2021, **11**:667495.

94. Ji J, Yang S, Zu L, Li Y, Li Y: **Deubiquitinating enzyme USP41 promotes lung cancer cell proliferation and migration**. *Thoracic cancer* 2021, **12**(7):1041-1047.

95. Huang M, Xiao J, Yan C, Wang T, Ling R: **USP41 promotes breast cancer via regulating RACK1**. *Annals of translational medicine* 2021, **9**(20):1566.

96. Hou K, Zhu Z, Wang Y, Zhang C, Yu S, Zhu Q, Yan B: **Overexpression and Biological Function of Ubiquitin-Specific Protease 42 in Gastric Cancer**. *PLoS One* 2016, **11**(3):e0152997.

97. Ye DX, Wang SS, Huang Y, Wang XJ, Chi P: **USP43 directly regulates ZEB1 protein, mediating proliferation and metastasis of colorectal cancer**. *J Cancer* 2021, **12**(2):404-416.

98. Lin F, Xie Z, Chang L, Li W, Wang L, Hou Y, Li L, Zhu J, Xia Y, He W *et al*: **USP43 promotes tumorigenesis through regulating cell cycle and EMT in breast cancer**. *International journal of clinical and experimental pathology* 2017, **10**(11):11014-11021.

99. Nishimura S, Oki E, Ando K, Iimori M, Nakaji Y, Nakashima Y, Saeki H, Oda Y, Maehara Y: **High ubiquitin-specific protease 44 expression induces DNA aneuploidy and provides independent prognostic information in gastric cancer**. *Cancer medicine* 2017, **6**(6):1453-1464.

100. Tian M, Zhu R, Ding F, Liu Z: **Ubiquitin-specific peptidase 46 promotes tumor metastasis through stabilizing ENO1 in human esophageal squamous cell carcinoma**. *Exp Cell Res* 2020, **395**(1):112188.

101. Pan B, Yang Y, Li J, Wang Y, Fang C, Yu FX, Xu Y: **USP47-mediated deubiquitination and stabilization of YAP contributes to the progression of colorectal cancer**. *Protein & cell* 2020, **11**(2):138-143.

102. Choi BJ, Park SA, Lee SY, Cha YN, Surh YJ: **Hypoxia induces epithelial-mesenchymal transition in colorectal cancer cells through ubiquitin-specific protease 47-mediated stabilization of Snail: A potential role of Sox9**. *Sci Rep* 2017, **7**(1):15918.

103. Silvestrini VC, Thomé CH, Albuquerque D, de Souza Palma C, Ferreira GA, Lanfredi GP, Masson AP, Delsin LEA, Ferreira FU, de Souza FC *et al*: **Proteomics analysis reveals the role of ubiquitin specific protease (USP47) in Epithelial to Mesenchymal Transition (EMT) induced by TGFβ2 in breast cells**. *Journal of proteomics* 2020, **219**:103734.

104. Naghavi L, Schwalbe M, Ghanem A, Naumann M: **Deubiquitinylase USP47 Promotes RelA Phosphorylation and Survival in Gastric Cancer Cells**. *Biomedicines* 2018, **6**(2).

105. Fraile JM, Campos-Iglesias D, Rodríguez F, Español Y, Freije JM: **The deubiquitinase USP54 is overexpressed in colorectal cancer stem cells and promotes intestinal tumorigenesis**. *Oncotarget* 2016, **7**(46):74427-74434.

106. Zhuang SM, Xie J, Zhen J, Guo LY, Hong ZD, Li FH, Xu DB: **The deubiquitinating enzyme ATXN3 promotes the progression of anaplastic thyroid carcinoma by stabilizing EIF5A2**. *Mol Cell Endocrinol* 2021, **537**.

107. Zhu RX, Gires O, Zhu LQ, Liu J, Li JJ, Yang H, Ju GD, Huang J, Ge WY, Chen Y *et al*: **TSPAN8 promotes cancer cell stemness via activation of sonic Hedgehog signaling**. *Nat Commun* 2019, **10**.

108. Zou HJ, Chen HY, Zhou Z, Wan Y, Liu ZH: **ATXN3 promotes breast cancer metastasis by deubiquitinating KLF4**. *Cancer Lett* 2019, **467**:19-28.

109. Li DY, Zhang T, Lai JJ, Zhang J, Wang T, Ling YF, He SQ, Hu ZW: **MicroRNA-25/ATXN3 interaction regulates human colon cancer cell growth and migration**. *Mol Med Rep* 2019, **19**(5):4213-4221.

110. Tao HH, Liao YX, Yan YJ, He ZW, Zhou JJ, Wang XH, Peng JP, Li SZ, Liu T: **BRCC3 Promotes Tumorigenesis of Bladder Cancer by Activating the NF-kappa B Signaling Pathway Through Targeting TRAF2**. *Front Cell Dev Biol* 2021, **9**.

111. Hu Y, Zhang Y, Ding M, Xu RS: **Long noncoding RNA TMPO-AS1/miR-126-5p/BRCC3 axis accelerates gastric cancer progression and angiogenesis via activating PI3K/Akt/mTOR pathway**. *J Gastroen Hepatol* 2021, **36**(7):1877-1888.

112. Zhang FF, Zhou Q: **Knockdown of BRCC3 exerts an anti-tumor effect on cervical cancer in vitro**. *Mol Med Rep* 2018, **18**(6):4886-4894.

113. Huang MW, Xiong H, Luo DL, Xu BR, Liu HL: **CSN5 upregulates glycolysis to promote hepatocellular carcinoma metastasis via stabilizing the HK2 protein**. *Exp Cell Res* 2020, **388**(2).

114. Wu BL, Pan YB, Liu GH, Yang T, Jin YX, Zhou FL, Wei YC: **MRPS30-DT Knockdown Inhibits Breast Cancer Progression by Targeting Jab1/Cops5**. *Front Oncol* 2019, **9**.

115. Liu GH, Yu MX, Wu BL, Guo S, Huang X, Zhou FL, Claret FX, Pan YB: **Jab1/Cops5 contributes to chemoresistance in breast cancer by regulating Rad51**. *Cell Signal* 2019, **53**:39-48.

116. Lu RQ, Hu XB, Zhou JM, Sun JJ, Zhu AZ, Xu XF, Zheng H, Gao X, Wang X, Jin HC *et al*: **COPS5 amplification and overexpression confers tamoxifen-resistance in ER alpha-positive breast cancer by degradation of NCoR**. *Nat Commun* 2016, **7**.

117. Zhang SH, Hong ZD, Chai Y, Liu ZQ, Du YY, Li Q, Liu QL: **CSN5 promotes renal cell carcinoma metastasis and EMT by inhibiting ZEB1 degradation**. *Biochem Bioph Res Co* 2017, **488**(1):101-108.

118. Zhang HQ, Zhong AL, Sun JJ, Chen MM, Xie SH, Zheng H, Wang YC, Yu YW, Guo L, Lu RQ: **COPS5 inhibition arrests the proliferation and growth of serous ovarian cancer cells via the elevation of p27 level**. *Biochem Bioph Res Co* 2017, **493**(1):85-93.

119. Jing C, Liu DD, Lai QC, Li LQ, Zhou MQ, Ye BB, Wu Y, Li H, Yue K, Wu YS *et al*: **JOSD1 promotes proliferation and chemoresistance of head and neck squamous cell carcinoma under the epigenetic regulation of BRD4**. *Cancer Cell Int* 2021, **21**(1).

120. Wu XW, Luo QY, Zhao PF, Chang W, Wang YT, Shu T, Ding F, Li B, Liu ZH: **JOSD1 inhibits mitochondrial apoptotic signalling to drive acquired chemoresistance in gynaecological cancer by stabilizing MCL1**. *Cell Death Differ* 2020, **27**(1):55-70.

121. Krassikova L, Zhang BX, Nagarajan D, Queiroz AL, Kacal M, Samakidis E, Vakifahmetoglu-Norberg H, Norberg E: **The deubiquitinase JOSD2 is a positive regulator of glucose metabolism**. *Cell Death Differ* 2021, **28**(3):1091-1109.

122. Zhang HH, Li C, Ren JW, Liu L, Du XH, Gao J, Liu T, Li SZ: **OTUB1 facilitates bladder cancer progression by stabilizing ATF6 in response to endoplasmic reticulum stress**. *Cancer Sci* 2021, **112**(6):2199-2209.

123. Zhao XD, Zhou M, Yang Y, Luo MJ: **The ubiquitin hydrolase OTUB1 promotes glioma cell stemness via suppressing ferroptosis through stabilizing SLC7A11 protein**. *Bioengineered* 2021, **12**(2):12636-12645.

124. Iglesias-Gato D, Chuan YC, Jiang N, Svensson C, Bao J, Paul I, Egevad L, Kessler BM, Wikstorm P, Niu YJ *et al*: **OTUB1 de-ubiquitinating enzyme promotes prostate cancer cell invasion in vitro and tumorigenesis in vivo**. *Mol Cancer* 2015, **14**.

125. Zhou Y, Wu JX, Fu X, Du WY, Zhou L, Meng XQ, Yu HY, Lin JX, Ye W, Liu JN *et al*: **OTUB1 promotes metastasis and serves as a marker of poor prognosis in colorectal cancer**. *Mol Cancer* 2014, **13**.

126. Yu SY, Zang WC, Qiu YC, Liao LM, Zheng XF: **Deubiquitinase OTUB2 exacerbates the progression of colorectal cancer by promoting PKM2 activity and glycolysis**. *Oncogene* 2022, **41**(1):46-56.

127. Liu GM, Guo W, Qin JJ, Lin ZL: **OTUB2 Facilitates Tumorigenesis of Gastric Cancer Through Promoting KDM1A-Mediated Stem Cell-Like Properties**. *Front Oncol* 2021, **11**.

128. Wan QY, Chen Q, Cai DG, Zhao Y, Wu XL: **OTUB2 Promotes Homologous Recombination Repair Through Stimulating Rad51 Expression in Endometrial Cancer**. *Cell Transplant* 2020, **29**.

129. Li J, Cheng DD, Zhu MX, Yu HJ, Pan Z, Liu L, Geng Q, Pan HY, Yan MX, Yao M: **OTUB2 stabilizes U2AF2 to promote the Warburg effect and tumorigenesis via the AKT/mTOR signaling pathway in non-small cell lung cancer**. *Theranostics* 2019, **9**(1):179-195.

130. Gong ZC, Li AC, Ding JC, Li Q, Zhang L, Li YP, Meng Z, Chen F, Huang JL, Zhou DW *et al*: **OTUD7B Deubiquitinates LSD1 to Govern Its Binding Partner Specificity, Homeostasis, and Breast Cancer Metastasis**. *Adv Sci* 2021, **8**(15).

131. Tang JN, Wu ZY, Tian ZL, Chen W, Wu GS: **OTUD7B stabilizes estrogen receptor alpha and promotes breast cancer cell proliferation**. *Cell Death Dis* 2021, **12**(6).

132. Lin DD, Shen Y, Qiao S, Liu WW, Zheng LS, Wang YN, Cui NP, Wang YF, Zhao SL, Shi JH: **Upregulation of OTUD7B (Cezanne) Promotes Tumor Progression via AKT/VEGF Pathway in Lung Squamous Carcinoma and Adenocarcinoma**. *Front Oncol* 2019, **9**.

133. Sun TS, Liu ZN, Bi FF, Yang Q: **Deubiquitinase PSMD14 promotes ovarian cancer progression by decreasing enzymatic activity of PKM2**. *Mol Oncol* 2021, **15**(12):3639-3658.

134. Jing C, Li XC, Zhou MQ, Zhang SC, Lai QC, Liu DD, Ye BB, Li LQ, Wu Y, Li H *et al*: **The PSMD14 inhibitor Thiolutin as a novel therapeutic approach for esophageal squamous cell carcinoma through facilitating SNAIL degradation**. *Theranostics* 2021, **11**(12):5847-5862.

135. Zhu R, Liu YS, Zhou HH, Li L, Li Y, Ding F, Cao XF, Liu ZH: **Deubiquitinating enzyme PSMD14 promotes tumor metastasis through stabilizing SNAIL in human esophageal squamous cell carcinoma**. *Cancer Lett* 2018, **418**:125-134.

136. Jing C, Duan YS, Zhou MQ, Yue K, Zhuo SS, Li XC, Liu DD, Ye BB, Lai QC, Li LQ *et al*: **yBlockade of deubiquitinating enzyme PSMD14 overcomes chemoresistance in head and neck squamous cell carcinoma by antagonizing E2F1/Akt/SOX2-mediated stemness**. *Theranostics* 2021, **11**(6):2655-2669.

137. Seo D, Jung SM, Park JS, Lee J, Ha J, Kim M, Park SH: **The deubiquitinating enzyme PSMD14 facilitates tumor growth and chemoresistance through stabilizing the ALK2 receptor in the initiation of BMP6 signaling pathway**. *Ebiomedicine* 2019, **49**:55-71.

138. Xu H, Yang XM, Xuan XF, Wu D, Zhang JR, Xu XC, Zhao YJ, Ma CP, Li DW: **STAMBP promotes lung adenocarcinoma metastasis by regulating the EGFR/MAPK signaling pathway**. *Neoplasia* 2021, **23**(6):607-623.

139. Okada R, Koshizuka K, Yamada Y, Moriya S, Kikkawa N, Kinoshita T, Hanazawa T, Seki N: **Regulation of Oncogenic Targets by miR-99a-3p (Passenger Strand of miR-99a-Duplex) in Head and Neck Squamous Cell Carcinoma**. *Cells-Basel* 2019, **8**(12).

140. Yu DJ, Qian J, Jin X, Li J, Guo CX, Yue XC: **STAMBPL1 knockdown has antitumour effects on gastric cancer biological activities**. *Oncol Lett* 2019, **18**(5):4421-4428.

141. Chen X, Shi HZ, Bi XG, Li YJ, Huang ZH: **Targeting the deubiquitinase STAMBPL1 triggers apoptosis in prostate cancer cells by promoting XIAP degradation**. *Cancer Lett* 2019, **456**:49-58.

142. Tangri A, Lighty K, Loganathan J, Mesmar F, Podicheti R, Zhang C, Iwanicki M, Drapkin R, Nakshatri H, Mitra S: **Deubiquitinase UCHL1 Maintains Protein Homeostasis through the PSMA7-APEH-Proteasome Axis in High-grade Serous Ovarian Carcinoma**. *Mol Cancer Res* 2021, **19**(7):1168-1181.

143. Kwan SY, Au-Yeung CL, Yeung TL, Rynne-Vidal A, Wong KK, Risinger JI, Lin HK, Schmandt RE, Yates MS, Mok SC *et al*: **Ubiquitin Carboxyl-Terminal Hydrolase L1 (UCHL1) Promotes Uterine Serous Cancer Cell Proliferation and Cell Cycle Progression**. *Cancers* 2020, **12**(1).

144. Fan Y, Hu D, Li D, Ma C, Tang Y, Tao Q, Deng L, Tang D: **UCHL3 promotes aerobic glycolysis of pancreatic cancer through upregulating LDHA expression**. *Clin Transl Oncol* 2021, **23**(8):1637-1645.

145. Ouyang LL, Yan B, Liu YT, Mao C, Wang M, Liu N, Wang ZL, Liu SP, Shi Y, Chen L *et al*: **The deubiquitylase UCHL3 maintains cancer stem-like properties by stabilizing the aryl hydrocarbon receptor**. *Signal Transduct Tar* 2020, **5**(1).

146. Zhang MH, Zhang HH, Du XH, Gao J, Li C, Shi HR, Li SZ: **UCHL3 promotes ovarian cancer progression by stabilizing TRAF2 to activate the NF-kappa B pathway**. *Oncogene* 2020, **39**(2):322-333.

147. Zhang JR, Xu H, Yang XM, Zhao YJ, Xu XC, Zhang L, Xuan XF, Ma CP, Qian WX, Li DW: **Deubiquitinase UCHL5 is elevated and associated with a poor clinical outcome in lung adenocarcinoma (LUAD)**. *J Cancer* 2020, **11**(22):6675-6685.

148. Liu D, Song ZX, Wang XY, Ouyang L: **Ubiquitin C-Terminal Hydrolase L5 (UCHL5) Accelerates the Growth of Endometrial Cancer via Activating the Wnt/beta-Catenin Signaling Pathway**. *Front Oncol* 2020, **10**.

149. Tian Z, D'Arcy P, Wang X, Ray A, Tai YT, Hu YG, Carrasco RD, Richardson P, Linder S, Chauhan D *et al*: **A novel small molecule inhibitor of deubiquitylating enzyme USP14 and UCHL5 induces apoptosis in multiple myeloma and overcomes bortezomib resistance**. *Blood* 2014, **123**(5):706-716.

150. Wu H, Xu HC, Jia DD, Li T, Xia LM: **METTL3-induced UCK2 m(6)A hypermethylation promotes melanoma cancer cell metastasis via the WNT/beta-catenin pathway**. *Ann Transl Med* 2021, **9**(14).

151. Wu YJ, Jamal M, Xie T, Sun JX, Song TB, Yin Q, Li JY, Pan S, Zeng XR, Xie SP *et al*: **Uridine-cytidine kinase 2 (UCK2): A potential diagnostic and prognostic biomarker for lung cancer**. *Cancer Sci* 2019, **110**(9):2734-2747.

152. Zhou QM, Jiang H, Zhang JL, Yu W, Zhou ZY, Huang PB, Wang J, Xiao ZY: **Uridine-cytidine kinase 2 promotes metastasis of hepatocellular carcinoma cells via the Stat3 pathway**. *Cancer Manag Res* 2018, **10**:6339-6355.

153. Huang SZ, Li J, Tam NL, Sun CJ, Hou YC, Hughes B, Wang ZK, Zhou Q, He XS, Wu LW: **Uridine-cytidine kinase 2 upregulation predicts poor prognosis of hepatocellular carcinoma and is associated with cancer aggressiveness**. *Mol Carcinogen* 2019, **58**(4):603-615.

154. Cai J, Sun XH, Guo H, Qu XY, Huang HT, Yu C, Wu HL, Gao YQ, Kong XN, Xia Q: **Non-metabolic role of UCK2 links EGFR-AKT pathway activation to metastasis enhancement in hepatocellular carcinoma**. *Oncogenesis* 2020, **9**(12).

155. Kim Y, Kim W, Song Y, Kim JR, Cho K, Moon H, Ro SW, Seo E, Ryu YM, Myung SJ *et al*: **Deubiquitinase YOD1 potentiates YAP/TAZ activities through enhancing ITCH stability**. *P Natl Acad Sci USA* 2017, **114**(18):4691-4696.

156. Miao DZ, Wang Y, Jia YH, Tong JX, Jiang SX, Liu LX: **ZRANB1 enhances stem-cell-like features and accelerates tumor progression by regulating Sox9-mediated USP22/Wnt/beta-catenin pathway in colorectal cancer**. *Cell Signal* 2022, **90**.

157. Zhang PJ, Xiao ZN, Wang SY, Zhang MT, Wei YK, Hang QL, Kim J, Yao F, Rodriguez-Aguayo C, Ton BN *et al*: **ZRANB1 Is an EZH2 Deubiquitinase and a Potential Therapeutic Target in Breast Cancer**. *Cell Rep* 2018, **23**(3):823-837.
